# Supplementary material for: Effects of horticultural therapy versus handiwork on anterior cingulate cortex activity in people with chronic low back pain: A randomized, controlled, cross-over, pilot study
Source: PLoS One. 2024 Dec 17;19(12):e0313920. doi: 10.1371/journal.pone.0313920 (PMC11651560; doi:10.1371/journal.pone.0313920)
Supplement: S1 File — (PDF) [file pone.0313920.s001.pdf]

"Comparative effects of horticultural therapy and handiwork on anterior cingulate cortex activation in people with chronic low back pain: a randomized controlled crossover pilot study (HORTICARE)."

Acronym: HORTICARE

RESEARCH PROTOCOL INVOLVING THE HUMAN PERSON
WITH MINIMAL RISKS AND CONSTRAINTS

Version N°2.0 of 10/25/2021

Project code: APHP191119 / IDRCB 2019-A02988-49

Coordinating investigator: Ms Alexandra ROREN
Service de Rééducation et de Réadaptation de l'Appareil Locomoteur and des Pathologies du Rachis
Department of Rehabilitation of the Musculoskeletal System and Spinal Pathologies
Hôpital Cochin
27 Rue du Faubourg Saint-Jacques, 75014 Paris
Tel: 01 58 41 13 71
alexandra.roren@aphp.fr

Scientific Director: Dr Christelle NGUYEN
Service de Rééducation et de Réadaptation de l'Appareil Locomoteur and des Pathologies du Rachis
Department of Rehabilitation of the Musculoskeletal System and Spinal Pathologies
Hôpital Cochin
27 Rue du Faubourg Saint-Jacques, 75014 Paris
Tel: 01 58 41 29 45
christelle.nguyen2@aphp.fr

Sponsor : Assistance Publique - Hôpitaux de Paris (AP-HP)
and by delegation: Direction de la Recherche Clinique et of Innovation (DRCI)
Saint-Louis Hospital
1, avenue Claude Vellefaux, 75010 Paris

Loaded structure research follow-up : Necker-Cochin Clinical Research Unit (URC)
Tarnier Hospital
89 rue d'Assas, 75006 Paris
DRCI-URC project coordinator: Laetitia PEAUDECERF
Tel: 01 58 41 12 13
laetitia.peaudecerf@aphp.fr

T ABLE OF CONTENTS

1 SYNOPTIC SUMMARY 5

2 SCIENTIFIC JUSTIFICATION OF RESEARCH 10

2.1 CURRENT STATE OF RESEARCH KNOWLEDGE 10
2.2 RESEARCH HYPOTHESES 11
2.3 DESCRIPTION OF THE POPULATION TO BE STUDIED AND JUSTIFICATION FOR ITS CHOICE 11
2.4 DESCRIPTION OF PROCEDURES PERFORMED IN ACCORDANCE WITH THEIR CURRENT USE 11
2.5 DESCRIPTION OF ACTS AND PROCEDURES ADDED BY SEARCH 13
2.6 SUMMARY OF FORESEEABLE AND KNOWN BENEFITS AND RISKS FOR RESEARCH SUBJECTS E 13

3 OBJECTIVES DE LA RECHERCHE 14

3.1 MAIN RESEARCH OBJECTIVE 14
3.2 SECONDARY RESEARCH OBJECTIVES 14

4 RESEARCH DESIGN 14

4.1 PRIMARY ENDPOINT 14

5 DESCRIPTION OF RESEARCH METHODOLOGY 16

5.1 EXPERIMENTAL DESIGN 16
5.2 NUMBER OF PARTICIPATING CENTERS 16
5.3 DESCRIPTION OF MEASURES TAKEN TO REDUCE AND AVOID BIAS 17

6 HOW WORKS 17

6.1 RESEARCH CALENDAR 18
6.2 SUMMARY TABLE OR DIAGRAM OF RESEARCH CHRONOLOGY 20
6.3 DISTINCTION BETWEEN CARE AND RESEARCH 21

7 SELECTION CRITERIA 21

7.1 INCLUSION CRITERIA 21
7.2 NON-INCLUSION CRITERIA 21
7.3 RECRUITMENT PROCEDURES 21

8 STOPPING RULES 22

8.1 CRITERIA AND PROCEDURES FOR PREMATURE TERMINATION OF A SUBJECT'S RESEARCH PARTICIPATION 22

9 EVALUATION OF EFFECTIVENESS 23

9.1 DESCRIPTION OF EVALUATION PARAMETERS OF EFFICACY 23
9.2 METHODS AND TIMETABLE FOR MEASURING, COLLECTING AND ANALYZING EVALUATION PARAMETERS OF EFFECTIVENESS 23

10 VIGILANCE 23

11 SPECIFIC RESEARCH COMMITTEES 24

11.1 STEERING COMMITTEE 24

12 DATA MANAGEMENT 24

12.1 DATA COLLECTION METHODS 24
12.2 RIGHT OF ACCESS TO SOURCE DATA AND DOCUMENTS 24
12.3 DATA PROCESSING AND STORAGE OF DOCUMENTS AND DATA 25
12.4 DATA OWNERSHIP 25

13 STATISTICAL ASPECTS 25

14 CONTROL AND QUALITY ASSURANCE 25

14.1 GENERAL ORGANIZATION 26

14.2 Observation notebook 26
14.3 Non-conformity management 26
14.4 Audit 26
14.5 Responsibilities of the Principal Investigator 26

15 ETHICAL AND LEGAL ASPECTS 27

15.1 Information and consent procedures for research subjects 27
15.2 Prohibition for the person to participate in other research or period of exclusion at the end of the research, if applicable 27
15.3 Registration in the national file of persons undergoing research involving the human person 27
15.4 Legal obligations 28
15.5 Request for an opinion from the Comite de protection des personnes CPP 28
15.6 Information from ANSM 28
15.7 Information technology and civil liberties 28
15.8 Search modifications 28
15.9 Final research report 28
15.10 Archiving 28

16 FINANCING AND INSURANCE 29

16.1 Source of financing 29
16.2 Insurance 29

17 PUBLICATION RULES 29

17.1 Mention of AP-HP affiliation for projects promoted by AP-HP 29
17.2 Mention of the AP-HP sponsor (DRCI) in the manuscript acknowledgments 30
17.3 Mention of the funder in the manuscript acknowledgments 30

18 BIBLIOGRAPHY 30

19 LIST OF ADDENDA 32

19.1 List of Investigators 32
19.2 APPENDICES 33
37

.

1 SYNOPTIC SUMMARY

Full title	**Comparative effects of horticultural therapy and handiwork on anterior cingulate cortex activation in people with chronic low back pain: a randomized controlled cross-over pilot study**
Acronym / Reference	**HORTICARE / APHP191119**
Coordinating investigator	Alexandra ROREN, rehabilitation manager<br>Service de Rééducation et de Réadaptation de l'Appareil Locomoteur and des Pathologies du Rachis<br>Hôpital Cochin<br>Tel: 01 58 41 13 71<br>alexandra.roren@aphp.fr
Scientific Manager	Dr Christelle NGUYEN<br>Service de Rééducation et de Réadaptation de l'Appareil Locomoteur and des Pathologies du Rachis<br>Hôpital Cochin<br>Tel: 01 58 41 29 45<br>christelle.nguyen2@aphp.fr
Developer	Assistance Publique - Hôpitaux de Paris
Scientific justification	Chronic low-back pain has considerable socio-economic repercussions. It is associated with physical and psychosocial de-conditioning.<br>An "experience of nature", even of short duration, has positive effects on the affective and cognitive factors involved in chronic pain. In the brain, the anterior cingulate cortex plays an important role in both pain and emotion. Exposure to a natural environment could reduce activation of the anterior cingulate cortex.
Objective and primary endpoint	<u>The main objective is to</u> evaluate the efficacy of horticultural therapy in reducing activation of the anterior cingulate cortex in people with chronic low back pain subjected to 2 sessions of 90 min of horticultural therapy and 2 sessions of 90 min of handiwork.<br><br><u>The primary endpoint</u> is the change in blood perfusion of the anterior cingulate cortex (in ml/100 g tissue/min) after the first exposure (MRI #2 - MRI #1) compared with the change after the second exposure (MRI #3 - MRI #1).
Objectives and secondary endpoints	<u>The secondary objectives are</u> :<br>- to compare the variation in: rumination score and dramatization score in patients with chronic low back pain after 2 sessions of 90 min of horticultural therapy versus 2 sessions of 90 min of handiwork.<br>-gather patient feedback on activities.<br><br><u>The quantitative secondary endpoints</u> will be :<br>- variation in rumination score measured by the self-administered Rumination Reflection Questionnaire (Trapnell and Campbell, 1999).

	- variation in dramatization score measured by the dramatization subscore of the self-administered Coping Strategy Questionnaire (Rosenstiel and Keefe, 1983; Irachabal et al., 2008).<br><br><u>Qualitative secondary endpoints</u> will be patients' opinions of the horticultural therapy and handiwork activities collected at the end of the intervention (during the final evaluation, after the end of the 2nd activity session during the last activity day of the 3rd week), using a standardized self-administered questionnaire comprising 4 closed questions and 1 open question to be answered in free text.<br><br>Other data will be collected (without comparative analysis):<br>- level of clinically significant symptoms of depression and anxiety assessed by the HAD self-administered scale.<br>- level of beliefs and expectations regarding treatment (at the end of 1st and 2nd exposure)<br>- level of fears and beliefs about work and physical activity assessed by the FABQ self-administered questionnaire<br>- variation in the level of activity limitation specific to low back pain as assessed by the Roland-Morris self-administered questionnaire<br>- variation in quality of life assessed by the SF-12 questionnaire.<br>Caregivers' opinions on the horticultural therapy and handiwork activities will be collected at the end of the intervention (during the final evaluation, after the end of the 2nd activity session during the last activity day of the 3rd week) by means of a standardized self-administered questionnaire comprising 2 closed questions and 1 open question to be answered in free text.
Experimental diagram	Single-center, randomized, controlled, cross-over pilot study using a mixed methodology (quantitative and qualitative).<br><br>The intervention comprises 3 stages (over 3 consecutive weeks):<br>- 1st stage (1st week) = 1st exposure: 2 x 90-minute activity sessions (horticulture or handiwork depending on randomization),<br>- 2ndstep (2nd week) = "wash out",<br>- 3rd stage (3rd week) = 2nd exposure: 2 sessions of 90 min of activity (horticulture or handiwork, different activity to the one practiced on 1st week).<br><br>Participants are their own controls and are randomized in a 1:1 ratio to receive a sequence of treatments involving the experimental intervention (horticulture) in either the 1st or 3rd stages, separated by a wash-out stage.<br>Therapists and participants are not blinded to the treatment sequence, but the neuroradiologist and biostatistician are.
Population concerned	Chronic low-back pain patients (current episode lasting > 3 months) for whom rehabilitation is indicated.
Inclusion criteria	- Adult patients ≥ 18 years,<br>- Chronic non-specific low back pain,<br>- Indication for rehabilitation,

	- Failure of 1st-line treatment,<br>- Up-to-date DTP (diphtheria tetanus poliomyelitis) vaccination,<br>- Patients able to walk 2 km,<br>- Membership of a social security scheme or beneficiary<br>- Signature of consent to participate.
Non-inclusion criteria	- Specific low back pain,<br>- MRI contraindications (metallic equipment, pacemaker, cochlear and intraocular metallic implants, current pregnancy, uncontrollable claustrophobia)<br>- Current work stoppage or work stoppage of more than 3 months in the last year,<br>- No paid employment<br>- Insufficient command of the French language,<br>- Participation in another research project on low back pain<br>- Patients under guardianship or curatorship,<br>- Patients on AME (State Medical Aid)
Acts or products to be researched	2 activities (horticulture and handiwork) are practiced by chronic low-back pain patients. Their effects on activation of the anterior cingulate cortex are compared.
Comparator group	The study design is cross-over, with participants being their own controls. The "comparator" intervention is handiwork.
Other acts or procedures added by search	Patients will undergo 3 MRI scans to measure blood perfusion in the anterior cingulate cortex :<br>- 1 MRI scan before 1st exposure (beginning of 1st week),<br>- 1 further MRI scan after 1st exposure (end of 1st week)<br>- 1 final MRI at the end of the procedure, after 2nd exposure (end of 3rd week)
Expected benefits for participants and the company	If successful, this study will enhance the therapeutic arsenal available to treat chronic low-back pain patients.<br>Horticultural therapy could be a precision non-pharmacological intervention for people with chronic low back pain, targeting both the neurobiological and physical aspects of exercise deconditioning.
Minimal risks and constraints added by research	The risks are minimal:<br>- related to the adverse events most frequently reported during moderate-intensity physical activity: pain, fatigue and musculoskeletal injuries.<br>- related to handling handiwork and gardening (injuries)<br>- related to MRI: presence of metal equipment, pregnancy, uncontrollable claustrophobia<br>- related to the patients' walk between Cochin and Sainte-Anne hospitals (patients will be accompanied by a member of the rehabilitation team during these journeys).<br><br>Patients have to travel to the Cochin hospital (4 times in 3 weeks), take part in craft and horticultural therapy

	sessions, undergo 3 brain MRI scans and walk to another hospital for this examination.
How it works in practice	Participants will be recruited from among patients seen in consultation by a doctor in the department or a doctor attached to the department. Participants will also be informed of the study by means of posters displayed in the Department Rehabilitation of the Musculoskeletal System and Spine Pathologies at Cochin Hospital, and in the waiting rooms of the private practices of doctors attached to the department. A patient included in the study will be able to call on his or her own network of acquaintances (recruitment based on the snowball effect). Participants will in groups of 4 in the rehabilitation department of Hôpital Cochin, and will perform the 1st or 3rd for 90 minutes twice a week:<br>The horticultural therapy activity (intervention) and twice a week on 1st or 3rd week, the handiwork activity (comparator).<br>3 MRI scans will be performed in the Diagnostic Neuroimaging Department at Hôpital Sainte-Anne.<br>MRI n°1 will be performed before the start of the 1st activity session (1st week: 1st exposure), MRI n°2 will be performed as soon as possible and no later than 3 hours after the end of the 3rd activity session, during the last day of activity of the 1st week (1st exposure), MRI n°3 will be performed as soon as possible and no later than 3 hours after the end of the 3rd activity session during the last day of activity of the 3rd week (3rd week: 2rd exposure). I<br>The self-administered Rumination Reflection Questionnaire and Coping Strategy Questionnaire will be completed by patients at 4 different times: before the start of the 1st activity session of the 1st week (1st exposure), after the end of the 2rd activity session of the 1st week (1stexposure), before the start of the 1st activity session of the 2nd week (2nd exposure) and at the end of the intervention, after the 2nd and last activity session of the 3rd week (2nd exposure). Patient feedback will be collected at the end of the intervention.
Number of subjects selected	Sample size is estimated at 16 participants. We predict a mean difference in variation in anterior cingulate cortex blood perfusion of 7 ml/100 g tissue/min with a standard deviation of 4, a power of 90%, an α risk of 5% and a potential lost to follow-up of around 20%.
Number of centers	One, the 2nd center is a "technical" center (performance of the primary endpoint evaluation procedure), with recruitment and treatment taking place in a single center.
Research calendar	- Inclusion period : 12 months<br>- Duration of participation (treatment + follow-up): 3 weeks<br>- Total duration: 13 months<br>Prohibition of participation in any other research on low back pain for as long as the research is ongoing.
Number of planned inclusions per center per month	2.7 / month.

	Single-center study (only 1 center involved in patient inclusion)
Statistical analysis	No intermediate analysis. Quantitative variables will be described by their means and standard deviation, and qualitative variables by their absolute and relative frequencies. Depending on the normality of the distribution of the quantitative variables of interest, they will be compared using a rank comparison test (Wilcoxon test) or a mean comparison test (Student's t-test for paired samples).
Source of financing	HUPC Research Department (APHP) and Fonds Luc (King Baudoin Foundation)

2 SCIENTIFIC JUSTIFICATION OF RESEARCH

2.1 CURRENT STATE OF RESEARCH KNOWLEDGE

2.1.1 On pathology

Non-specific low back pain is the leading cause of years lived with disability worldwide (Disease GBD, Lancet 2017). It becomes chronic when its duration of evolution exceeds 12 weeks and then represents the leading cause of disability at work (Palazzo et al., 2016). The network of biological, psychological and social contributors to chronic low back pain is complex. Within this network, physical and psychic deconditioning to exercise is central and is a preferred therapeutic target of multidisciplinary exercise retraining programs.
Chronic low back pain is associated with physical deconditioning, defined as a reduction in spinal mobility and muscle performance.

On reference strategies/procedures

To take account of the multifactorial and complex nature of low back pain, the biopsychosocial model has been favoured in recent decades.
It has led to the emergence of standardized intensive multidisciplinary programs based on exercise retraining (ERT), combining education, exercise therapy, physical activity, occupational therapy and psychological and social rehabilitation (Rozenberg et al., 2012). Functional restoration programs aimed at combating physical deconditioning characterized by reduced spinal mobility and muscular performance of the spinal muscles, include stretching exercises for the sub-pelvic muscles and muscle strengthening (Mayer et al., 1985).

Exercise improves pain and function in chronic low-back pain (Hayden et al., 2005, Koes et al., 2006). However, its benefits diminish if it is not continued on a regular basis (Taimela et al., 2000). The enjoyment of physical exercise and the possibility of integrating it into daily life are important factors in adherence (McArthur et al., 2014). Gardening is well suited to the physical treatment of chronic low back pain, since it involves working on spinal flexibility (during activities such as weeding and leaf collection, etc.), strength and endurance of spinal and limb muscles (carrying bags of soil, carrying tools, handling watering cans, etc.) and proprioceptive adjustments (walking on more or less stable ground, digging on more or less hard ground, etc.). What's more, gardening is an activity with tangible objectives and results. Associated with the notion of pleasure, it can be practised in a group, fostering social ties. Gardening is suitable for the long term, and can even be practised in town (community gardens...).

Chronic pain is associated with affective disorders such as anxiety and depression (Landa et al., 2012). Pain sensations are in fact modulated by affective and emotional state (Sullivan et al., 2001). Within the pain neuro-matrix, the anterior cingulate cortex plays an important role in encoding pain and associated emotions (Vogt, 2005). It also acts as an interface between cognition and emotion (Dum et al., 2016). In major depressive pathologies, connections between the default brain network and the anterior cingulate cortex are strengthened and perfusion of the anterior cingulate cortex is increased (Hamilton et al., 2015). Activation of the anterior cingulate cortex is particularly linked to rumination, defined as the intrusion of involuntary and difficult-to-control thoughts (Kuehner and Weber, 1999, Nolen-Hoeksema, 2000).

Several studies suggest an impact of a "nature experience", even of short duration, on several physiological parameters: brain activity, autonomic nervous system, hormonal and immune system (Franco et al., 2017, Hansen et al., 2017). Simply seeing plants is said to induce a decrease in prefrontal cortex activity measured on MRI, stabilization of autonomic nervous activity measured by heart rate regularity and a feeling of well-being (White et al., 2013, Ikei et al., 2014, Park et al., 2016). The physiological mechanisms behind these effects remain incompletely elucidated. Several theories are proposed to support the effects of nature on health and well-being. According to attention restoration theory, a natural environment produces stimuli that enable the subject to decentralize and thus reduce attentional fatigue linked to cognitive tasks (Kaplan and Kaplan, 1999). According to stress recovery theory, an extension of the biophilic hypothesis, man is innately connected to nature. Nature is therefore a necessary building block for development, balance and adaptation to stress (Ulrich, 1983). In a randomized controlled trial published in 2015 in the journal PNAS, conducted in 38 healthy volunteers, Bratman and colleagues compared the effect of a 90-

min walk in nature (forest) with a 90-min walk in an urban environment (city) on activation of the anterior cingulate cortex, assessed by means of MRI blood perfusion variation, and on rumination, assessed by means of a self-questionnaire (Bratman et al., 2015). The authors showed that neural activation in the anterior cingulate cortex and rumination scores were lower in the group of healthy volunteers exposed to nature (Bratman et al., 2015). A cross-over study showed beneficial effects of a nature walk on mood and particularly rumination in patients with major depressive disorder (Berman et al., 2012). A randomized controlled trial showed that patients in the sub-acute phase of a stroke adhered widely to a nature-based activity program (Palsdottir et al., 2020). The impact of a "nature experience" on chronic low back pain has been little studied (Hyunju et al., 2019). A non-randomized controlled study showed the effectiveness of adding 7 horticultural therapy sessions to a standardized pain management program, on the health status, anxiety and coping strategies of chronic pain patients (fibromyalgia and chronic low back pain) (Verra et al., 2012).

Through its dual nature-experimental and physical components, horticultural therapy could be a precision non-pharmacological intervention in people with chronic low back pain, targeting both the neurobiological and physical aspects of exercise deconditioning. Horticultural therapy was introduced in October 2017 into the multidisciplinary exercise retraining programs dedicated to chronic low back pain in our department. However, its effects have not yet been evaluated.

2.2 Research hypotheses

We hypothesize that horticultural therapy may reduce activation of the anterior cingulate cortex. Its effects could be mediated by dual exposure to both nature and physical activity. The pilot dimension of this study is in line with the methodological structuring of complex interventions recommended by the Medical Research Council.

2.3 Description of the population to be studied and justification for its choice

Participants will be recruited from chronic low-back pain sufferers for whom rehabilitation is indicated.

2.4 Description of procedures performed in accordance with their current use

Horticultural and handiwork activities have been part of the routine care of chronic low-back pain patients in our department for many years (25 years for the handiwork workshop and 3 years for the horticultural workshop). The horticultural and handiwork activities are supervised by experienced professionals (occupational therapists) (> 10 years' experience), well-versed in running therapeutic workshops.

Participants will be in groups of 4, to perform for 90 minutes 2 times a week, 2 consecutive days, on 1st or 3rd week: the horticulture activity (intervention) and 2 times a week on 1st or 3rd week, the handiwork activity (comparator). The HANDIWORK activity was chosen as comparator because the level of physical activity proposed is comparable to that of gardening (comparable approximate energy expenditure value, (Ainsworth et al., 2000) and exposure to nature is nil. The 2 activities involve handling tasks: lifting an object off the ground and carrying it, and forward-leaning standing postures reproducing the conditions of use of the spine in multiple occupational and daily activities, and soliciting the spinal muscles (deficient in chronic low-back pain patients, Demoulin et al., 2007).

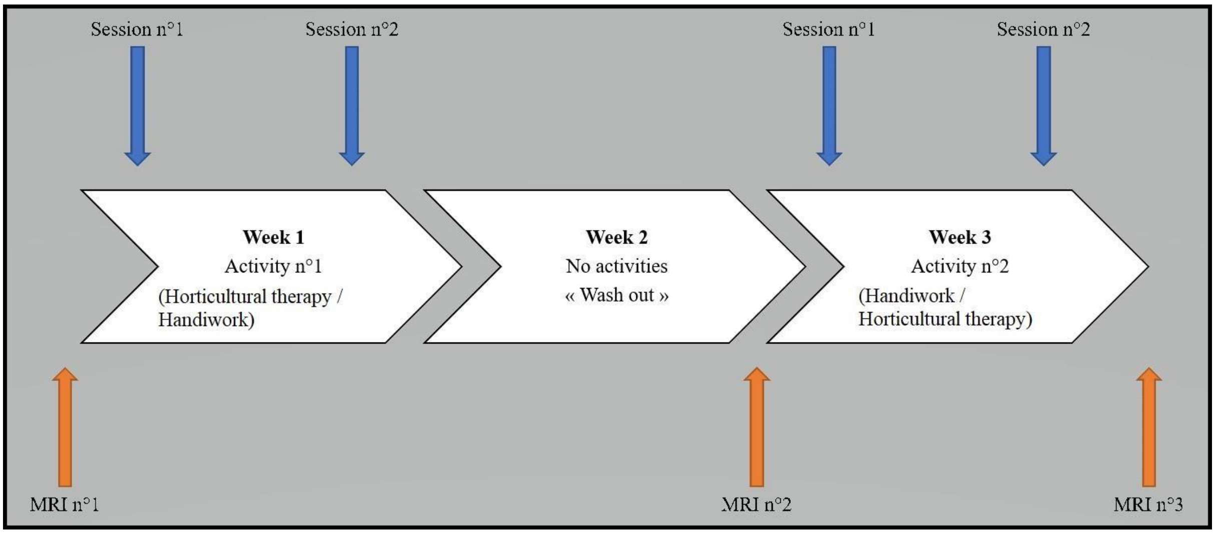

Contents of horticultural and HANDIWORK workshops :

Horticultural workshop: this workshop will be supervised by an experienced occupational therapist in collaboration with the hospital's gardeners.
The tasks performed will be :

- Handling tasks (moderate loads (maximum load: 8 kg), standing): carrying tools (rake, spade, wheelbarrow, pruning shears...), carrying bags of potting soil, handling full watering cans (approx. 20 min).

- gardening tasks (standing, leaning forward (forward lunge), crouching or kneeling, etc.): weeding, picking up plants from the ground and from raised containers, digging, cutting, planting, watering, mulching, picking fruit and vegetables from the vegetable garden (approx. 70 min.)

The content of gardening tasks depends on the season and the gardener's planning of activities. In the event that weather conditions make outdoor work difficult, horticultural activities in the greenhouse: handling, planting, cutting, making cuttings... are possible (standing and sitting up high). The greenhouse was financed by the Trophée patients AP-HP 2018, "coup de cœur du jury" prize).

Do-it-yourself workshop: this workshop will be supervised by an experienced occupational therapist.
The tasks performed will be :

- handling tasks (moderate loads (maximum load: 8 kg), in standing position): transport (wood, etc.) between the technical services and the occupational therapy room (the 2 buildings are approximately 200 m apart, duration of these tasks, approximately 20 min)
- actual handiwork tasks standing, leaning forward (forward lunge), crouching or kneeling): taking measurements, cutting wood (1 cm thick boards) with a hand saw, assembling, finishing (approx. 70 min.)

The aim of these handiwork tasks is to build a small wooden cabinet to store occupational therapy equipment.

To consolidate the description of activities, we used the TIDieR item list.

2.5 Description of acts and procedures added by search

The procedures added by the research will be brain MRIs. These will be performed at Hôpital St Anne on a Canon Galan 3T XGO clinical MRI unit. Patients will be positioned supine in the MRI ring, with their arms at their sides, and protected against noise pollution (earplugs). A 32-channel surface antenna will be installed for image acquisition.
The total duration of the exam is approximately 35 minutes. It will consist of 3 sequences:

- high-resolution T1-weighted image for anatomical reference (Canon sequence: 3D FAST FE, equivalent to MPRAGE)
- ASL, perfusion-weighted image (Canon sequence: ASTAR)
- PD, proton density-weighted image for quantitative CBF measurement (Canon sequence: ASTAR, with long TR)

No injection of contrast medium will be performed.

2.6 Summary of foreseeable and known benefits and risks for research subjects e

With its dual experimental nature and physical component, horticultural therapy could be a precision non-pharmacological intervention for people with chronic low back pain, targeting both the neurobiological and physical aspects of exertional deconditioning. Therapeutic horticulture also has the advantage of being practicable over the long term.

The risks are minimal:

- related to the adverse events most frequently reported during moderate-intensity physical activity: pain, fatigue and musculoskeletal injuries.

- related to handling handiwork and gardening tools (skin injuries)

- if known contraindications are respected: pacemaker, cochlear and intraocular metallic implants, these risks are low. These contraindications will have been ruled out at the inclusion visit.

- related to patients' walking distance (approx. 1km) between Hôpital Cochin and Hôpital Sainte-Anne (patients will be accompanied by a member of the rehabilitation team during these journeys).

- for the horticultural workshop, patients will be equipped with safety shoes, gardening gloves and, depending on weather conditions, a waterproof coat. For the handiwork activity, patients will be equipped with safety shoes, protective gloves and goggles (for cutting tasks). The department already has and uses this equipment, which is cleaned/disinfected between each use.
Thanks to the use of specially adapted equipment, and the constant supervision of the activity by caregivers who explain and demonstrate the movements to be carried out, and assist patients encountering difficulties, we have not had to deplore any injuries (apart from a few rare blisters and splinters when patients were not wearing gloves).

Given the current health situation, and in the absence of new recommendations, all patients will be equipped with disposable masks, hands will be systematically washed with SHA before starting work, and gloves will be put on. We will also ensure physical distancing between patients and between patients and staff.

3 OBJECTIVES DE LA RECHERCHE

3.1 Main research objective

The main objective was to evaluate the efficacy of horticultural therapy in reducing activation of the anterior cingulate cortex in people with chronic low back pain subjected to 2 sessions of 90 min of horticultural therapy versus 2 sessions of 90 min of HANDIWORK.

3.2 Secondary research objectives

The secondary objectives are :

- to compare the change in rumination score and dramatization score in patients with chronic low back pain after 2 sessions of 90 min of horticultural therapy versus 2 sessions of 90 min of handiwork.

- gather patient feedback on activities.

4 RESEARCH DESIGN

4.1 Primary endpoint

The primary endpoint is the change in anterior cingulate cortex activity measured by the change in blood perfusion in ml/100 g tissue/min.

The change in blood perfusion in the anterior cingulate cortex after the first exposure (MRI #2 - MRI #1) will be compared with the change after the second exposure (MRI #3 - MRI #1).

MRI n°1 will be performed before the start of the 1st activity session of the 1st treatment sequence (1st week: 1st exposure), MRI n°2 will be performed as soon as possible and no later than 3 hours after the end of the 2rd activity session, during the last day of activity of the 1st week (1st exposure), MRI n°3 will be performed as soon as possible and no later than 3 hours after the end of the 2nd activity session of the 2nd treatment sequence, during the last day of activity of the 3rd week (3rd week: 2nd exposure). The patient's activity schedule will be adjusted to ensure that the 3-hour delay between the end of the activity and the MRI is respected for each patient.

Illustration of a treatment sequence

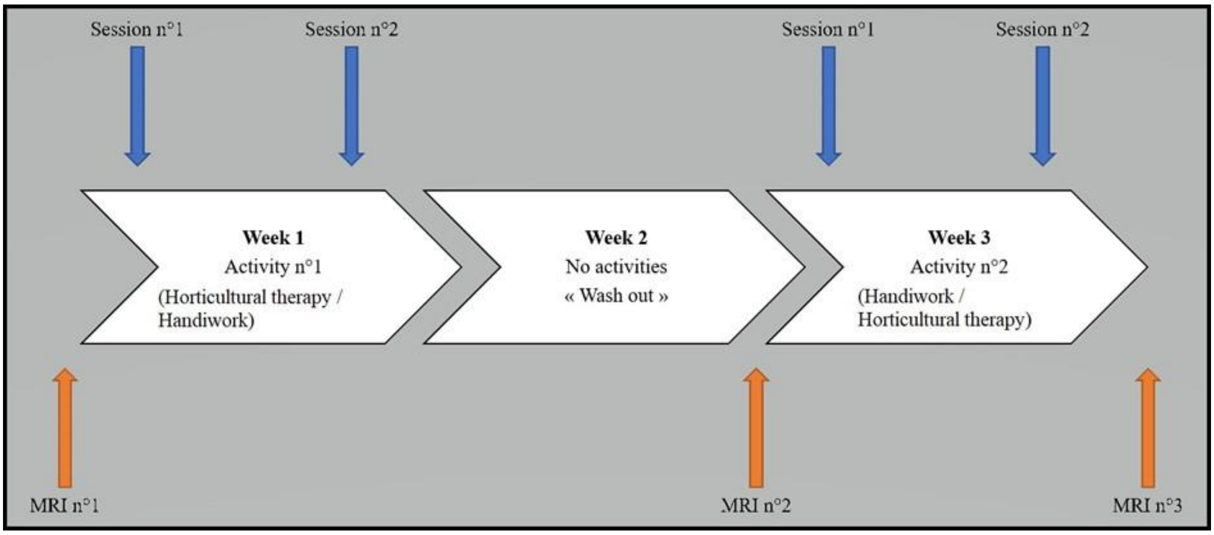

The quantitative secondary endpoints were :

- the variation (after 2 sessions of 90 min of horticultural therapy versus 2 sessions of 90 min of handiwork) in the level of rumination measured using the self-administered 'Rumination Reflection Questionnaire' (Appendix D) validated in English and translated into French with the help of a professional translator,

- the change (after 2 sessions of 90 min horticultural therapy versus 2 sessions of 90 min handiwork) in the level of dramatization measured by the dramatization sub-score of the self-administered Coping Strategy Questionnaire (Appendix E).

The self-administered 'Rumination Reflection Questionnaire' and 'Coping strategy questionnaire' will be completed by patients:

- before the start of the 1st activity session (1st week: 1st exhibition),
- after the end of the 2nd activity session, during the last activity day of the 1st week (1st exhibition)
- after the end of the 2nd activity session on the last activity day of the 3rd week (3rd week: 2nd exposure).

The qualitative secondary endpoints were patients' opinions of the horticultural therapy and handiwork activities collected at the end of the intervention (during the final evaluation, after the end of the 2nd activity session on the last activity day of the 3rd week) using a standardized self-administered questionnaire with closed questions.

Responses will be expressed using an 11-class Likert scale from 0 to 10) and 1 open-ended question, the answers to which will be expressed in free text (Appendix H).

The thematic analysis of the text will be carried out by 2 independent reviewers.

Other data will be collected (without comparative analysis):

- level of beliefs and expectations regarding treatment (5, no beliefs and expectations, and 45, maximum beliefs and expectations) (Appendix B).

- level of clinically significant depressive symptoms assessed by the depression subscale of the self-administered Hospital Anxiety Depression HADd scale (0, no symptoms and 21, maximum symptoms) (Appendix G).

-level of clinically significant anxiety symptoms assessed by the anxiety subscale of the HADa self-administered scale (0, no symptoms and 21, maximum symptoms) (Appendix G).

- level of fears and beliefs assessed by the self-administered Fear-Avoidance Beliefs Questionnaire (FABQ) work: (0, no fears and beliefs and 24, maximum fears and beliefs) and physical activity: (0, no fears and beliefs and 42, maximum fears and beliefs) (Appendix F).

- variation in the level of activity limitation specific to low back pain, as assessed by the Roland-Morris self-administered questionnaire (0, no limitation, 24, maximum limitation) (Appendix C).
- change in quality of life as assessed by the SF-12 questionnaire (increase in score means improvement in quality of life) (Appendix A).

These questionnaires will be completed by patients:

before the start of the 1st activity session (1st week: 1st exhibition),

after the end of the 2nd activity session, on the last activity day of the 1st week (1st exhibition)

after the end of the 2nd activity session, on the last activity day of the 3rd week (2nd exhibition).

Activities performed during the treatment abstention period will be collected prior to the second treatment sequence (3rd week: 2nd exposure).

Caregivers' opinions on the horticultural therapy and handiwork activities will be collected at the end of the intervention (during the final evaluation, after the end of the 2nd activity session on the last activity day of the 3rd week) by means of a standardized self-administered questionnaire comprising 2 closed questions (for 1 of these questions, answers will be expressed using an 11-

class Likert scale from 0 to 10) and 1 open question whose answer will be expressed in free text (Appendix I).

Illustration of a treatment sequence

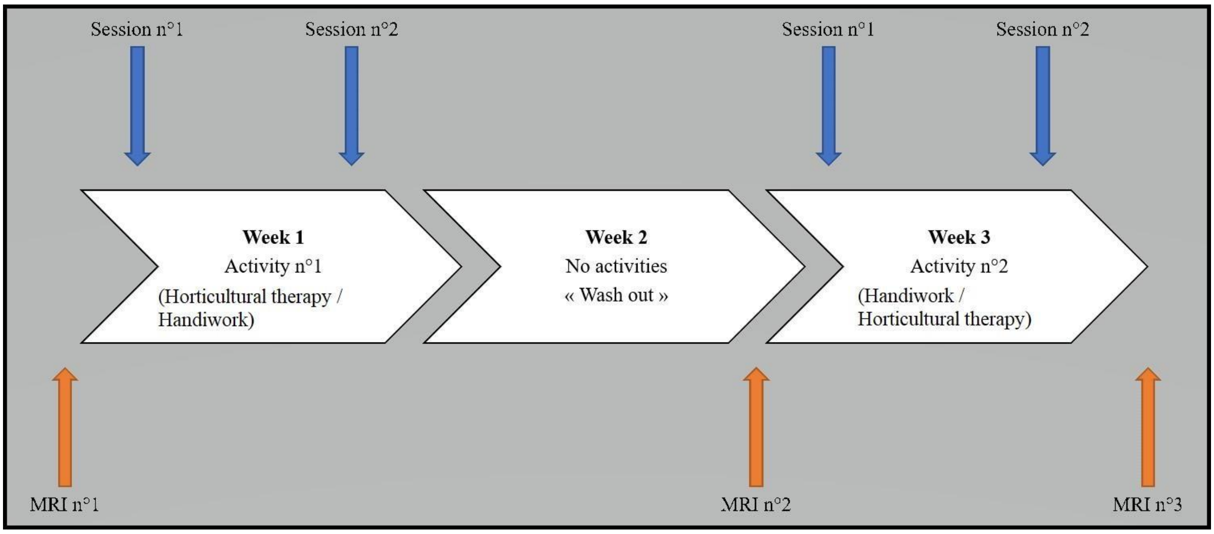

5 DESCRIPTION OF RESEARCH METHODOLOGY

5.1 Experimental design

Pilot randomized controlled cross-over study in the Department of Re-education and Rehabilitation of the Musculoskeletal System and Rachis Pathologies at Hôpital Cochin (Pr François RANNOU) using a mixed methodology (quantitative and qualitative).

Participants will be their own controls and will be randomized in a 1:1 ratio to receive a sequence of treatments involving the experimental intervention (horticulture) in either stage 1 or stage 3, separated by a wash-out stage.

Therapists and participants will not be blinded to the treatment sequence, but the neuroradiologist and biostatistician will be.

5.2 Number of participating centers

There is only 1 recruiting center (Service de Rééducation et de Réadaptation de l'Appareil Locomoteur et des Pathologies du Rachis, Hôpital Cochin).

However, another center is associated with this study, the Diagnostic Neuroimaging Department of the Hôpital Sainte-Anne (Pr C. OPPENHEIM), where the MRI scans will be performed and analyzed.

- **Recruiting centers**

Service de Rééducation et de Réadaptation de l'Appareil Locomoteur et des Pathologies du Rachis, Hôpital Cochin.

- **Non-recruiting centers**

MRI scans will be carried out in the Diagnostic Neuroimaging Department at Hôpital Sainte-Anne (Pr C. OPPENHEIM), 1 km from Hôpital Cochin. Patients will be accompanied on foot by staff from the rehabilitation department.

MRI n°1 will be performed on the 1st day of treatment, before the start of the 1st activity session. MRIs n°2 and n°3 will be performed at the end of the last activity session, at the end of weeks 1 and 3rd respectively.

5.3 Description of measures taken to reduce and avoid bias

5.3.1 Subject identification

For the purposes of this research, subjects will be identified as follows:
center no. (3 numeric positions) - person selection order no. in center (3 numeric positions) - initial surname - initial first name

This reference is unique and will be kept for the duration of the research.

5.3.2 Randomization

The treatment sequence will be randomly allocated using an interface implemented in Cleanweb software, and will take place in 3 stages over 3 consecutive weeks.

Depending on the group allocated, the treatment sequence will begin with either the experimental intervention (horticulture) or the control intervention (handiwork).

The 3-week period was chosen to minimize the influence of the natural evolution of symptoms on the primary endpoint.

In addition, since each participant is his or her own control, his or her exposome should vary little over this period. Significant changes in exposome will be collected using a checklist. The 1-week wash-out period seems sufficient to allow a return to basal levels of activation of the anterior cingulate cortex in response to each participant's habitual exposome.

6 HOW WORKS

Persons whose consent is sought	Who informs and obtains the person's consent	When is the person informed?	When is the person's consent obtained?
• the person carrying out the research ;	• *the principal investigator, co-investigator*	• when selecting "telephone screening<br>• and again at the inclusion visit	• consent is obtained at the time of the inclusion visit, after a minimum reflection period of one week

Example of the treatment sequence starting with the control intervention :

- Stage 1 (1st week: 1st exposure period: do-it-yourself): on the 1er day of treatment, before the start of the 1st activity session: participants will undergo a brain MRI in the Diagnostic Neuroimaging department at Hôpital Sainte-Anne (Pr Oppenheim) (MRI n°1). During this week, participants will have 2 activity sessions (crafts) lasting 90 minutes, led by an occupational therapist with experience of this activity with people suffering from chronic low back pain, and taking place in the occupational therapy room (in the basement of the Hardy building). At the end of the last activity session (handiwork) of the 1st week, participants will undergo a new brain MRI (MRI n°2) in the Diagnostic Neuroimaging department of the Hôpital Sainte-Anne. The time between the end of the activity and the MRI will be a maximum of 3 hours. Patients will be accompanied on their journey between the Cochin and Sainte-Anne hospitals by a nurse from the Cochin hospital rehabilitation department.

- Stage 2 (week 2: wash-out period): no activities will be organized during this week. Participants will continue with their usual daily activities.

- 3rd stage (3rd week: 2nd exposure period: horticulture): participants will have 2 sessions of 90-minute horticultural therapy activities, led by an occupational therapist with experience of this activity with chronic low-back pain sufferers, and taking place in the green outdoor premises of Hôpital Cochin. The department has purchased a greenhouse to enable the horticultural

activity to continue whatever the weather conditions. At the end of the last horticultural workshop, participants will undergo a final brain MRI (MRI n°3). The time between the end of the activity and the MRI will be a maximum of 3 hours. Patients will be accompanied on their journey from Hôpital Cochin to Hôpital Sainte-Anne by a nurse from the Cochin rehabilitation department.

Illustration of a treatment sequence

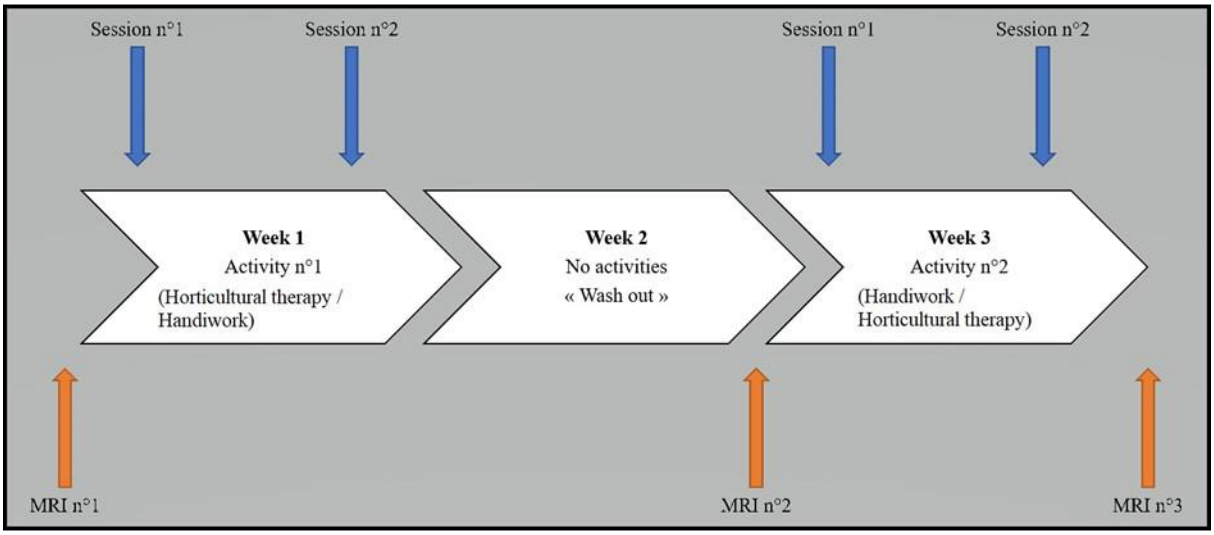

6.1 Research calendar

Maximum time between selection and inclusion	1 month
Length of inclusion period	12 months
Duration of subject participation, of which :	
Duration of intervention	3 weeks
Follow-up time	No follow-up outside intervention
Total research time	13 months

6.1.1 Selection visit

The doctor asks the patient if he or she agrees to be contacted by telephone to participate in a study... If the patient agrees, the doctor puts him or her on a list.

Selection: patients on the list are called by the investigator (or a Clinical Study Technician (CST)). During this telephone interview, patients are informed of the objectives and content of the study. If patients agree to take part, an inclusion visit is scheduled (within a minimum of one week (cooling-off period) and a maximum of one month).

6.1.2 Inclusion visit

The investigator responsible for inclusion will check the inclusion criteria, inform the patient of the objectives of the study using an information note, collect the patient's written consent, collect the data, randomize the patient using a website dedicated to the study (e-CRF Cleanweb, Télémédecine company), and inform the patient of his or her appointments according to the allocated allocation group.

Collection of socio-demographic and professional characteristics :

- surname, first name, date of birth (partially entered, only month and year of birth will be shown), postal address and e-mail address,

- size, weight
- professional information:
    - level of education, last occupation
    - Satisfaction with current job
    - Past work stoppages due to low back pain
    - Number and duration of days off work since onset of low back pain non-specific
    - Number of days off work in the last year
    - Low back pain related to a work accident :
        - if yes: date of accident :
    - Low back pain linked to an occupational disease
        - if yes: date of onset of occupational illness :

- Sports activities :
    - Volume of weekly sports activities (end date, if applicable)
    - Current impact of low back pain on your sporting activities

Collection of clinical characteristics :

- Date of onset of low back pain
- Start date of current episode
- Taking painkillers and NSAIDs (non-steroidal anti-inflammatory drugs)

Self-administered questionnaires: at the beginning of 1st exposure, before the start of the 1st activity session.
-Rumination level assessed by the Rumination Reflection Questionnaire (0: no rumination and 60: maximum rumination)
-Level of dramatization assessed by the dramatization sub-score of the self-administered Coping Strategies Questionnaire (CSQ) (0, no dramatization, 20, maximum dramatization).

Collection of MRI characteristics (MRI no. 1): blood perfusion of the anterior cingulate cortex (in ml/100 g tissue/min): at the start of the 1st exposure, before the start of the 1st activity session.

Sequences used :

- high-resolution T1-weighted image for anatomical reference (Canon sequence: 3D FAST FE, equivalent to MPRAGE)
- ASL, perfusion-weighted image (Canon sequence: ASTAR)
- PD, proton density-weighted image for quantitative CBF measurement (Canon sequence: ASTAR, with long TR)

Injection of contrast media: none

6.1.3 Research follow-up visits

No follow-up outside intervention

Self-administered questionnaires: at the end of 1st exposure and at the beginning of 2nd exposure
-Rumination level assessed by the Rumination Reflection Questionnaire (0: no rumination and 60: maximum rumination)
-Level of dramatization assessed by the dramatization sub-score of the self-administered Coping Strategies Questionnaire (CSQ) (0, no dramatization, 20, maximum dramatization).

Collection of MRI features (MRI no. 2): blood perfusion of the anterior cingulate cortex (in ml/100 g tissue/min): at the end of the 1st exposure, after the third activity session.

6.1.4 End of research visit

End of research equals end of intervention

Self-administered questionnaires: at the end of the 2nd exhibition, after the last activity session:

-Rumination level assessed by the Rumination Reflection Questionnaire (0: no rumination and 60: maximum rumination)

-Level of dramatization assessed by the dramatization sub-score of the self-administered Coping Strategies Questionnaire (CSQ) (0, no dramatization and 20, maximum dramatization).

Collection of MRI features (MRI no. 3): blood perfusion of the anterior cingulate cortex (in ml/100 g tissue/min): at the end of the 2nd exposure, after the end of the last activity session.

Sequences used :

- high-resolution T1-weighted image for anatomical reference (Canon sequence: 3D FAST FE, equivalent to MPRAGE)
- ASL, perfusion-weighted image (Canon sequence: ASTAR)
- PD, proton density-weighted image for quantitative CBF measurement (Canon sequence: ASTAR, with long TR)

Injection of contrast media: none

No medical follow-up is planned at the end of the study. Patients will be monitored as planned by the referring physician.

Group analysis :

- Correction of ASL data movements (with FSL's BASIL toolbox)
- Quantification of cerebral blood flow = CBF (with FSL's BASIL toolbox)
- Calculation of the ASL image transformation on the anatomical image (with ANTs software)
- Calculation of anatomical image transformations on a standard space, e.g. ICBM152 (with ANTs software)
- Applying these transformations to IVC maps
- Extraction of IVC values from an atlas (DKT) and statistical analysis of standardized IVC maps.

6.2 Summary table or diagram of research chronology

	Selection S-4 to S-1	Inclusion and randomization S 0	Start of intervention S+2 to S+4	End of 1st exhibition S+3 to S+5	Start of 2nd exhibition S+5 to S+7	End of 2nd exhibition: S+6 to S+8
Information	✓	✓				
Written consent		✓				
Randomization		✓				
Clinical examination [1]		✓				
Paraclinical examinations (and questionnaires)[2]			✓	✓	✓	✓

[1]Clinical examination:

Collection of clinical characteristics

- date of onset of low back pain
- start date of current episode
- taking painkillers and NSAIDs (non-steroidal anti-inflammatory drugs)

[2]Para-clinical examinations :

-Self-administered questionnaires: at the start of the 1st exposure, before the start of the 1st activity session.

-Rumination level assessed by the Rumination Reflection Questionnaire (0: no rumination and 60: maximum rumination)

-Level of dramatization assessed by the dramatization subscore of the self-administered Coping Strategies Questionnaire (CSQ) (0, no dramatization, 20, maximum dramatization).

-Collection of MRI features (MRI no. 1): blood perfusion of the anterior cingulate cortex (in ml/100 g tissue/min)

6.3 Distinction between care and research

Research interventions	Acts, procedures and treatments related to care	Acts, procedures added by search
**Consultations**	NA	Inclusion visit
**Imaging**	NA	MRI and self-administered questionnaires:<br>- at the start of the 1st exhibition, before the start of the 1st activity session.<br>- at the end of the 1st show and at the beginning of the 2nd show<br>- at the end of the 2nd exhibition, after the last activity session.

7 SELECTION CRITERIA

7.1 Inclusion criteria

- Adult patients ≥ 18 years,
- Chronic non-specific low back pain (current episode lasting ≥ 3 months),
- Indication for rehabilitation
- Failure of 1st line treatment,
- Up-to-date DTP (diphtheria tetanus poliomyelitis) vaccination,
- Patients able to walk 2 km,
- Membership of a social security scheme or beneficiary
- Signature of consent to participate.

7.2 Non-inclusion criteria

- Specific low back pain,
- MRI contraindications (metallic equipment, pacemaker, cochlear and intraocular metallic implants, current pregnancy, uncontrollable claustrophobia)
- Current work stoppage or work stoppage of more than 3 months in the last year,
- No paid employment
- Insufficient command of the French language,
- Participation in another research project on low back pain
- Patients under guardianship or curatorship,
- Patients on AME (State medical Aid).

7.3 Recruitment procedures

Justification of recruitment capacity with regard to the number of subjects to be included ;

A difference of 7 ml/100 g tissue/min (SD=4) between perfusion changes in the anterior cingulate cortex after the 2 horticultural therapy sessions and after the 2 handiwork sessions is estimated to be clinically relevant (Bratman et al., 2015). Given this expected difference, a risk α of 5%, a power of 90%, and a potential lost to follow-up of 20%, we planned to include a total of 16 patients.

Participants will be recruited from among patients seen in consultation by a doctor in the department or a doctor attached to the department. Participants will also be informed of the study by means of posters displayed in the Department of Re-education and Rehabilitation of the Musculoskeletal System and Spine Pathologies at Cochin Hospital, and in the waiting rooms of the private practices of doctors attached to the department (Appendix J and K). A patient included in the study will be able to call on his or her own network of acquaintances (recruitment based on the snowball effect).in the department It is during a consultation that the physician (-investigator) asks the patient if he or she agrees to be contacted by telephone about a study.

	Number of subjects
Total number of subjects selected	16
Number of recruiting centers	1
Inclusion period (months)	12
Number of subjects / center	16
Number of subjects / center / month	**1,3**

8 STOPPING RULES

8.1 Criteria and procedures for premature termination of a subject's research participation

- Any subject may stop participating in research at any time, for any reason.
- Subject lost to follow-up: the subject's whereabouts are unknown. The investigator must make every effort to reconnect with the subject (and document this in the source file) in order to know at least whether the subject is alive or dead.
- The investigator may temporarily or permanently discontinue a subject's participation in the research for any reason that has an impact on the subject's safety or is in the subject's best interest.
- In the event of premature termination of the subject's participation in the research, his or her data may be used until the date of withdrawal of consent.

In the event of premature termination of a subject's research, or withdrawal of consent, data concerning the subject collected prior to premature termination may be used.

- The observation book should list the various reasons for discontinuing participation in the research:
  - ☐ Undesirable effect
  - ☐ Other medical problems
  - ☐ Subject's personal reasons
  - ☐ Explicit withdrawal of consent.

- Arrangements for replacing these persons, if necessary

No replacement subjects planned.

- Stopping all or part of the search

The AP-HP promoter reserves the right to definitively suspend inclusions at any time, should it prove that inclusion targets are not being met.

9 EVALUATION OF EFFECTIVENESS

9.1 Description of evaluation parameters of efficacy

The primary endpoint is the change in anterior cingulate cortex activity measured by the change in blood perfusion in ml/100 g tissue/min. The change in blood perfusion of the anterior cingulate cortex after the first exposure (MRI n°2 - MRI n°1) will be compared with the change after the second exposure (MRI n°3 - MRI n°1). MRI n°1 will be performed before the start of the 1st activity session (1st week: 1st exposure), MRI n°2 will be performed after the end of the 2nd activity session, during the last day of activity of the 1st week (1st exposure), MRI n°3 will be performed after the end of the 2nd activity session during the last day of activity of the 3rd week (3rd week: 2nd exposure).

9.2 Methods and timetable for measuring, collecting and analyzing evaluation parameters of effectiveness

Illustration of a treatment sequence

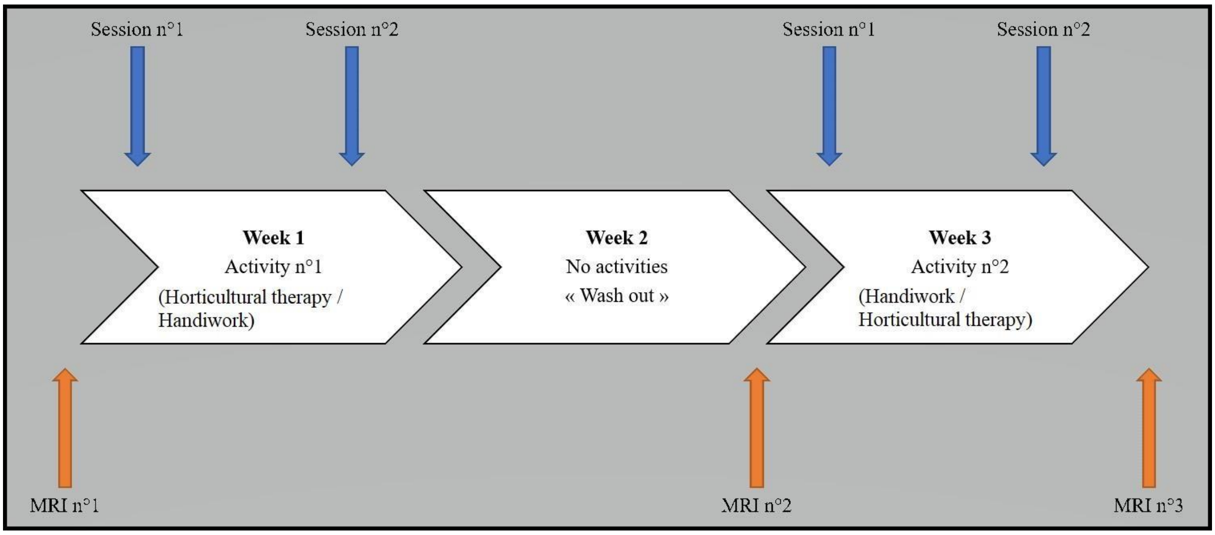

Collection of MRI characteristics (MRI no. 1): blood perfusion of the anterior cingulate cortex (in ml/100 g tissue/min): at the start of the 1st exposure, before the start of the 1st activity session.

Collection of MRI features (MRI no. 2): blood perfusion of the anterior cingulate cortex (in ml/100 g tissue/min): at the end of 1st exposure, after the second activity session.

Collection of MRI features (MRI no. 3): blood perfusion of the anterior cingulate cortex (in ml/100 g tissue/min): at the end of the 2nd exposure, after the end of the last activity session.

10 VIGILANCE

In the context of this study, adverse events (serious or otherwise) are not notifiable to the sponsor.

If such events occur, they are related to the pathology or its therapeutic management in the context of care (horticultural and handiwork sessions) and are not related to research. Serious adverse

events will be reported within the framework of the vigilance system set up for the practice under research (radiovigilance).

The sponsor undertakes to inform the competent authority and the Committee for the Protection of Individuals without delay of the new facts defined in 12° of article R. 1123-46 and, where applicable, of the measures taken.

11 SPECIFIC RESEARCH COMMITTEES

11.1 Steering Committee

The steering committee will be composed of :

The coordinating investigator Alexandra ROREN, the scientific manager, Doctor Christelle NGUYEN, the methodologists in charge of the project: Doctor Hendy ABDOUL, the managers of the URC/ CIC Paris Descartes Necker/Cochin or the Project referent of the URC/CIC Paris Descartes Necker/Cochin.

The role of the steering committee is to :

-Define the general organization and progress of the research, and coordinate information.

-Determine the methodology at the outset, and decide during the course of the research what to do in the event of unforeseen circumstances.

-Monitor the search process.

12 DATA MANAGEMENT

12.1 Data collection methods

Clinical and paraclinical signs will be collected and entered into an electronic data collection notebook (e-CRF CleanWEB), with access restricted by a login and password, unique to each investigating physician in charge of a patient. The data entered will be anonymized and secured by encryption during data transfer. The sponsor will use the information concerning the participant in accordance with the objectives of the study or for any additional scientific research.

12.2 Right of access to source data and documents

- Data access

In accordance with GCP :

- the sponsor is responsible for obtaining the agreement of all parties involved in the research to guarantee direct access to all research sites, source data, source documents and reports for quality control and audit purposes by the sponsor,

- investigators will make available to the persons responsible for monitoring and quality control, in the event of an audit of research involving the human person, the documents and individual data strictly necessary for this control, in accordance with the legislative and regulatory provisions in force

- Source documents

Source documents, defined as any original document or object that can be used to prove the existence or accuracy of data or facts recorded during the course of the research, will be kept for 15 years by the investigator, or by the hospital in the case of hospital medical records.

All data comes from the medical record, except for questionnaires, which are collected directly from patients and entered into e-CRF.

- Data confidentiality

Those responsible for quality control in research involving the human body (article L.1121-3 of the French Public Health Code), will take all necessary precautions to ensure the confidentiality of information relating to the research, to the persons involved, and in particular to their identity, and to the results obtained.

These people, like the investigators themselves, are bound by professional secrecy (under the conditions defined by articles 226-13 and 226-14 of the French penal code).

During or at the end of research involving the human person, data collected on subjects and transmitted to the sponsor by the investigators (or any other specialist) will be rendered non-identifying.

Under no circumstances may the names or addresses of the persons concerned appear in clear text.

Only the initials of the surname and first name will be recorded, together with a coded number specific to the research indicating the order of inclusion of subjects.

Similarly, if the results of this study are published, the participant's identity will remain confidential.

These data may also be transmitted to the French health authorities or to other entities of the promoter, under conditions that ensure their confidentiality.

The sponsor will ensure that each person taking part in the research has given his or her written consent for access to individual data concerning him or her and strictly necessary for the quality control of the research.

12.3 Data processing and storage of documents and data

- Identification of the data controller and data processing location

All data will be entered into an electronic CleanWeb data collection notebook (e-CRF). The Unité de Recherche Clinique Paris Centre will be responsible for research data management and database management (Manager: Pr Jean-Marc TRELUYER, Deputy Manager: Dr Hendy ABDOUL).

- Data entry

Non-identifying data will be entered electronically via a web browser.

12.4 Data ownership

The AP-HP is the owner of the data and no use or transmission to a third party may be made without its prior agreement.

13 STATISTICAL ASPECTS

Intention-to-treat study

Quantitative variables will be described by their means and standard deviation, and qualitative variables by their absolute and relative frequencies.

Depending on the normality of the distribution of the quantitative variables of interest, they will be compared using a rank comparison test (Wilcoxon test) or a mean comparison test (Student's t-test for paired samples).

14 CONTROL AND QUALITY ASSURANCE

14.1 General organization

The sponsor must ensure the safety and respect of those who have agreed to take part in the research. They must set up a quality assurance system to monitor the progress of the research in the investigating centers.

To this end, the promoter will define a strategy for opening centers and may, if necessary, set up a data quality control system.

14.1.1 Center opening strategy

The strategy for opening the centers will be determined before the research begins.

14.2 Observation notebook

Electronic CRF :

All information required by the protocol must be recorded in the observation notebooks. Data should be collected as they are obtained, and recorded explicitly in these notebooks. Any missing data should be coded.
This electronic case report form will be set up in each center using an Internet data collection medium. Investigators will be provided with a document to help them use this tool.

The investigator is responsible for the accuracy, quality and relevance of all data entered. Moreover, when data is entered, it is immediately checked for consistency. In this respect, the investigator must validate any value modification in the CRF. These modifications are subject to an audit trail. A justification may be included as a comment.

A hard copy will be requested at the end of the study, authenticated (dated and signed) by the investigator. The original of this document will be archived by the sponsor. A copy of the authenticated document must be archived by the investigator.

14.3 Non-conformity management

Any event arising from non-compliance with the protocol, standard operating procedures or applicable laws and regulations by an investigator or any other person involved in the conduct of research must be reported to the sponsor.

These non-conformities will be managed in accordance with the sponsor's procedures.

14.4 Audit

Investigators agree to accept quality assurance audits by the sponsor and inspections by competent authorities. All data, documents and reports are subject to audit and regulatory review without prejudice to medical confidentiality.

An audit may be carried out at any time by persons mandated by the sponsor and independent of those in charge of the research. Its purpose is to ensure the quality of the research, the validity of its results and compliance with the law and regulations in force.

Those conducting and supervising the research agree to comply with the sponsor's requirements for an audit.

The audit can be applied to all stages of research, from protocol development to publication of results, and to the classification of data used or produced as part of the research.

14.5 Responsibilities of the Principal Investigator

Before starting the research, each investigator will provide the research sponsor's representative with an updated personal curriculum vitae, dated less than one year and signed, including his/her RPPS number. The CV should include previous research participation and training related to clinical research.

Each investigator undertakes to comply with legislative and regulatory obligations and to conduct the research in accordance with regulations, respecting the terms of the declaration of Helsinki in force.

The principal investigator at each participating center will sign an undertaking of responsibility (DRCI-type document), which will be given to the sponsor's representative.
The investigators and their collaborators will sign a delegation of functions form specifying the role of each and will provide their CVs.

15 ETHICAL AND LEGAL ASPECTS

15.1 Information and consent procedures for research subjects

In accordance with article L1122-1-1 of the French Public Health Code, no research involving minimal risk and minimal constraints on the human body may be carried out on a person without his or her free, informed and express consent, after the person has been given the information specified in article L. 1122-1 of the same code.

A sufficient period of reflection will be allowed between the time the person is informed and the time he or she signs the consent form.

The person's free, informed and written consent is obtained by the principal investigator, or by a physician representing him or her, or by the qualified person before the person is included in the research.

The information note and a copy of the consent form, dated and signed by the person undergoing the research and by the investigator or the physician representing him or her or the qualified person, are given to the person prior to participation in the research.

In addition, the investigator must specify in the patient's medical record that the patient is participating in the research, how consent is obtained and how the information required to obtain consent is provided. The investigator keeps the original copy of the consent form, dated and signed.

15.2 Prohibition for the person to participate in other research or period of exclusion at the end of the research, if applicable

During the period of participation in the research, the subject may not participate in another research protocol concerning chronic low back pain. The subject may not take part in any other research protocol involving the human body without having discussed the matter with the physician who is treating him/her as part of the research.

At the end of the subject's participation, no exclusion period is defined in this research.

15.2.1 **Reimbursement of expenses**

No travel expenses reimbursed

15.2.2 **Compensation**

A 50-euro allowance has been set aside for patients to compensate for research-related constraints.

15.3 Registration in the national file of persons undergoing research involving the human person

Not planned

15.4 Legal obligations

Assistance publique hôpitaux de Paris (AP-HP) is the promoter of this research, and the Direction de la Recherche Clinique et de l'Innovation (DRCI) is responsible for its missions, in accordance with article L.1121-1 of the French Public Health Code.

Assistance Publique - Hôpitaux de Paris reserves the right to interrupt the research at any time for medical or administrative reasons; in this eventuality, the investigator will be notified.

15.5 Request for an opinion from the Comité de protection des personnes CPP

For interventional research involving minimal risk and constraints, the AP-HP, as promoter, obtains a favorable opinion from the relevant CPP prior to its implementation, within the scope of its competence and in accordance with the legislative and regulatory provisions in force.

15.6 Information from ANSM

The AP-HP promoter will send the CPP's favorable opinion and the protocol summary to ANSM for information.

15.7 Information technology and civil liberties

The computer file used for this research is implemented in accordance with French (amended Data Protection Act) and European (General Data Protection Regulation -RGPD.

- Commitment to comply with MR 001 "Reference Methodology

This research falls within the scope of the "Reference Methodology for the Processing of Personal Data Implemented in the Context of Healthcare Research" (MR-001 modified). The AP-HP, promoter of the research, has signed a commitment to comply with this "Reference Methodology"

15.8 Search modifications

Any substantial changes made to the protocol by the coordinating investigator must be forwarded to the sponsor for approval. Once approved, the sponsor must obtain a favorable opinion from the CPP prior to implementation.

The information note and consent form may be revised if necessary, particularly in the event of substantial changes to the research or the occurrence of adverse effects.

15.9 Final research report

The final report on the research involving the human person, mentioned in article R1123-67 of the CSP, is drawn up and signed by the sponsor and the investigator. A summary of the report, drawn up in accordance with the competent authority's reference plan, must be sent to the competent authority within one year of the end of the research, corresponding to the end of the participation of the last person involved in the research.

15.10 Archiving

Documents specific to human research involving minimal risk and minimal constraints will be archived by the investigator and sponsor for 15 years after the end of the research.

This indexed archive includes :

- A sealed investigator's envelope containing an original copy of all the information notes and consent forms signed by everyone at the center who participated in the research;
- A sealed envelope for the sponsor containing a copy of all the information notes and consent forms signed by all the people at the center who took part in the research;

- Research" binders for the Investigator and Sponsor including (non-exhaustive list):
    - successive versions of the protocol (identified by version number and date), its appendices
    - PPC opinions
    - correspondence,
    - the inclusion list or register,
    - research-specific appendices
    - the final research report.
- Data collection documents.

16 FINANCING AND INSURANCE

16.1 Source of financing

HUPC Research Department (APHP) and Fonds Luc (King Baudoin Foundation)

16.2 Insurance

In accordance with article L.1121-10 of the French Public Health Code, insurance policies must cover the civil liability of the sponsor and any other parties involved, and cover the financial consequences of claims arising from research involving the human body.

The Sponsor, for the entire duration of the research, takes out insurance covering its own civil liability as well as that of any physician involved in carrying out the research. It also guarantees full compensation for the harmful consequences of the research for the person taking part and his or her beneficiaries, unless it can prove that the damage is not attributable to its own fault or to that of any other party involved, without being able to invoke the act of a third party or the voluntary withdrawal of the person who had initially agreed to take part in the research.

Assistance Publique- Hôpitaux de Paris (AP-HP) has taken out insurance with HDI-GLOBAL SE through BIOMEDIC-INSURE, covering its civil liability as well as that of all participants (doctors or staff involved in the research), in accordance with article L.1121-10 of the CSP.

17 PUBLICATION RULES

The APHP must be mentioned in the affiliations of the author(s) of publications resulting from this research, and must mention the AP-HP promoter (DRCI) and the source of funding if your project is the result of a call for tenders (e.g. national or regional PHRC), and send us a copy (see below for details of affiliation and mention of promoter and funders).

17.1 Mention of AP-HP affiliation for projects promoted by AP-HP

- If an author has several affiliations, the order in which the institutions are cited (AP-HP, University, INSERM...) is not important.
- However, if the research is funded under an internal AP-HP call for tenders, the first affiliation should be "AP-HP".
- Each of these affiliations must be identified by an address separated by a semicolon (;
- The institution AP-HP must appear under the acronym "**AP-HP**" first in the address followed precisely by : **AP-HP**, hospital, department, city, zip code, France

17.2 Mention of the AP-HP sponsor (DRCI) in the manuscript acknowledgments

"The sponsor was Assistance Publique - Hôpitaux de Paris (Direction de la Recherche Clinique et de l'Innovation)"

17.3 Mention of the funder in the manuscript acknowledgments

If this was an internal AP-HP tender, please specify: "The study was funded by a grant from Assistance Publique - Hôpitaux de Paris".

This search will be registered on http://clinicaltrials.gov/

18 BIBLIOGRAPHY

1. Trapnell PD, Campbell JD. Private self-consciousness and the five-factor model of personality: distinguishing rumination from reflection. J Pers Soc Psychol. 1999;76(2):284-304.
2. Rosenstiel AK, Keefe FJ. The use of coping strategies in chronic low back pain patients: relationship to patient characteristics and current adjustment. Pain 1983;17:33-44.
3. Irachabal S, Koleck M, Rascle N, Bruchon-Schweitzer M. [Pain coping strategies: French adaptation of the coping strategies questionnaire (CSQ-F)]. Encephale. 2008;34(1):47-53.
4. Disease GBD, Injury I, Prevalence C. Global, regional, and national incidence, prevalence, and years lived with disability for 328 diseases and injuries for 195 countries, 1990-2016: a systematic analysis for the Global Burden of Disease Study 2016. Lancet 2017;390(10100):1211-59.
5 Palazzo C, Ravaud JF, Papelard A, Ravaud P, Poiraudeau S. The burden of musculoskeletal conditions. PLoS One. 2014;4;9(3):e90633.
6. Rozenberg S, Foltz V, Fautrel B. Treatment strategy for chronic low back pain. Joint Bone Spine. 2012 Dec;79(6):555-9. doi: 10.1016/j.jbspin.2012.09.003.
7. Mayer TG, Gatchel RJ, Mayer H et al. A prospective two-year study of functional restoration in industrial low back injury. An objective assessment procedure. JAMA. 1987;258(13):1763-7.
8. Hayden JA, van Tulder MW, Malmivaara A, Koes BW. Exercise therapy for treatment of non-specific low back pain. Cochrane Database Syst Rev. 2005(3):CD000335.
9. Koes, B.W.; van Tulder, M.W.; Thomas, S. Diagnosis and treatment of low back pain.Br. Med. J.2006;332,1430-1434
10. Taimela S, Diederich C, Hubsch M, Heinricy M. The role of physical exercise and inactivity in pain recurrence and absenteeism from work after active outpatient rehabilitation for recurrent or chronic low back pain: a follow-up study. Spine (Phila Pa 1976). 2000;15;25(14):1809-16.
11. McArthur D, Dumas A, Woodend K, Beach S, Stacey D. Factors influencing adherence to regular exercise in middle-aged women: a qualitative study to inform clinical practice. BMC Womens Health. 2014 26;14:49.
12. Landa A, Peterson BS, Fallon BA. Somatoform pain: A developmental theory and translational research review. Psychosom Med 2012;74:717-27.
13. Sullivan MJ, Thorn B, Haythornthwaite JA, et al. Theoretical perspectives on the relation between catastrophizing and pain. Clin J Pain 2001;17:52-64.
14. Vogt BA. Pain and emotion interactions in subregions of the cingulate gyrus. Nat Rev Neurosci 2005;6:533-544.
15 Dum RP, Levinthal DJ, Strick PL. Motor, cognitive, and affective areas of the cerebral cortex influence the adrenal medulla. Proc Natl Acad Sci U S A. 2016;30;113(35):9922-7.
16. Hamilton JP, Farmer M, Fogelman P, Gotlib IH. Depressive rumination, the default-mode network, and the dark matter of clinical neuroscience. Biol Psychiatry 2015;15;78(4):224-30.
17. Kuehner C, Weber I. Responses to depression in unipolar depressed patients: An investigation of Responses to depression in unipolar depressed patients: An investigation of Nolen-Hoeksema's response styles theory. Psychol Med 1999;29(6): 1323-1333.

18. Nolen-Hoeksema S. The role of rumination in depressive disorders and mixed anxiety/depressive symptoms. J Abnorm Psychol 2000;109(3):504-511.
19. Franco LS, Shanahan DF, Fuller RA. A Review of the Benefits of Nature Experiences: More than Meets the Eye. Int. J. Environ. Res. Public Health 2017,14,864.
20. Hansen MM; Jones R, Tocchini K. Shinrin-Yoku (Forest Bathing) and Nature Therapy: A State-of-the-Art Review. Int. J. Environ. Res. Public Health 2017,14,851.
21. White MP, Alcock I, Wheeler BW, Depledge MH. Would you be happier living in a greener urban area? A fixed-effects analysis of panel data. Psychol Sci 2013;24(6): 920-928.
22. Ikei H, Song C, Igarashi M, Namekawa T, Miyazaki, Y. Physiological and psychological relaxing effectsof visual stimulation with foliage plants in high school students. Adv. Hortc. Sci. 2014, 28, 111-116.
23. Park S.A, Song C, Choi J.Y, Son K.C. Miyazaki, Y. Foliage plants cause physiological and psychological relaxation as evidenced by measurements of prefrontal cortex activity and profile of mood states. HortScience 2016, 51, 1308-1312.
24. Kaplan R. and Kaplan S.. The experience of nature: A psychological perspective. 1989 Cambridge University Press, Cambridge, UK.
25. Ulrich R.S. Aesthetic and affective response to natural environment. 1983 p. 85-125. In: I.Altman and J.F.Wohlwill (eds.). Behavior and the natural environment. Springer US, Plenum Press, New York, NY.
26. Bratman GN, Hamilton JP, Hahn KS, Daily GC, Gross JJ. Nature experience reduces rumination and subgenual prefrontal cortex activation. Proc Natl Acad Sci U S A. 2015 14;112(28):8567-72.
27. Berman MG, Kross E, Krpan KM, Askren MK, Burson A, Deldin PJ, Kaplan S, Sherdell L, Gotlib IH, Jonides J. Interacting with nature improves cognition and affect for individuals with depression. J Affect Disord 2012;140(3):300-305.
28. Pálsdóttir AM, Stigmar K, Norrving B, Petersson IF, Åström M, Pessah-Rasmussen H. The nature stroke study; NASTRU: A randomized controlled trial of nature-based post-stroke fatigue rehabilitation. J Rehabil Med. 2020;52(2):jrm00020.
29. Verra ML, Angst F, Beck T, et al. Horticultural therapy for patients with chronic musculoskeletal pain: results of a pilot study [published correction appears in Altern Ther Health Med. 2012 Nov-Dec;18(6):79. Verra, Martin L [corrected to Verra, M L]]. Altern Ther Health Med. 2012;18(2):44-50.
30. Hyunju J, Chorong S, Yoshifumi M. Physiological Benefits of Viewing Nature: A Systematic Review of Indoor Experiments; Int J Environ Res Public Health. 2019;16(23): 4739.
31. Ainsworth BE, Haskell WL, Whitt MC, Irwin ML, Swartz AM, Strath SJ, O'Brien WL, Bassett DR Jr, Schmitz KH, Emplaincourt PO, Jacobs DR Jr, Leon AS. Compendium of physical activities: an update of activity codes and MET intensities. Med Sci Sports Exerc. 2000 Sep;32(9 Suppl):S498-504.
32 Demoulin C., Crielaard J.-M., Vanderthommen M. Spinal muscle evaluation in healthy individuals and low-back-pain patients: a literature review. Joint Bone Spine. 2007;74(1):9-13. doi: 10.1016/j.jbspin.2006.02.013

19 LIST OF ADDENDA

19.1 List of Investigators

Search location coordinates	Title	First name Last name	Telephone / e-mail
Hôpital Cochin, Department of Rehabilitation of the Musculoskeletal System and Spinal Pathologies		Alexandra ROREN	Tel: 01 58 41 13 71 alexandra.roren@aphp.fr
	MCU-PH	Christelle NGUYEN	Tel: 01 58 41 29 45 christelle.nguyen2@aphp.fr
	PU-PH	François RANNOU	Tel: 01 58 41 25 35 francois.rannou@aphp.fr
	Dr.	Marie-Martine LEFEVRE-COLAU	Tel: 01 58 41 25 42 marie-martine.lefevre-colau@aphp.fr
Hôpital Cochin, Radiology Department B	PU-PH	Antoine FEYDY	Tel: 01 58 41 24 83 antoine.feydy@aphp.fr
St Anne's Hospital, Diagnostic Neuroimaging Department	PU-PH	Catherine OPPENHEIM	Tel: 01 45 65 82 42 c.oppenheim@ch-sainte-anne.fr

19.2 APPENDICES

APPENDIX A

ANNEXE A

QUESTIONNAIRE DE QUALITÉ DE VIE SF-12

Les questions qui suivent portent sur votre santé, telle que vous la percevez. Vos réponses permettront de suivre l'évolution de votre état de santé et de savoir dans quelle mesure vous pouvez accomplir vos activités courantes.
Répondez à toutes les questions en suivant les indications qui vous sont données. En cas de doute, répondez de votre mieux.

1. Dans l'ensemble, pensez-vous que votre santé est :
(Cochez une seule réponse)

- Excellente ....☐
- Très bonne ....☐
- Bonne ....☐
- Médiocre ....☐
- Mauvaise ....☐

➢ Voici une liste d'activités que vous pouvez avoir à faire dans votre vie de tous les jours. Pour chacune d'entre elles indiquez si vous êtes limité(e) en raison de votre état de santé actuel.

(Cochez une seule réponse par ligne)

	**très limité(e)**	**un peu limité(e)**	**pas du tout limité(e)**
**2. Efforts physiques modérés** tels que déplacer une table, passer l'aspirateur, jouer aux boules	☐ ☐	☐	
**3.** Monter **plusieurs étages** par l'escalier	☐	☐	☐

➢ **Au cours de ces <u>4 dernières semaines</u>, et en raison de votre <u>état physique</u>,**

(Cochez une seule réponse par ligne)

	**OUI**	**NON**
**4.** Avez-vous **accompli moins** de choses que vous auriez souhaité	☐	☐

This document is the property of DRCI / APHP. Any reproduction is strictly forbidden.

5. Avez-vous du arrêter de faire certaines choses ☐ ☐

➢ **Au cours de ces <u>4 dernières semaines</u>, et en raison de votre <u>état émotionnel</u> (comme vous sentir triste, nerveux(se) ou déprimé(e))**

(Cochez une seule réponse par ligne)

	OUI	NON
**6.** Avez-vous **accompli moins** de choses que vous auriez souhaité	☐	☐
**7.** Avez-vous eu des **difficultés** à faire ce que vous aviez à faire **avec autant de soin et d'attention que d'habitude**	☐	☐

8. Au cours de ces <u>4 dernières semaines</u>, dans quelle mesure vos douleurs physiques vous ont-elles limité(e) dans votre travail ou vos activités domestiques ?

(Cochez une seule réponse)

- Pas du tout.. ☐
- Un petit peu... ☐
- Moyennement .... ☐
- Beaucoup .... ☐
- Énormément .... ☐

➢ Les questions qui suivent portent sur comment vous vous êtes senti(e) <u>au cours de ces 4 dernières semaines</u>. Pour chaque question, veuillez indiquer la réponse qui vous semble la plus appropriée.

➢ **Au cours de ces <u>4 dernières semaines</u>, y a-t-il eu des moments où :**

(Cochez une seule réponse par ligne)

	**en permanence**	**très souvent**	**souvent**	**quelquefois**	**rarement**	**jamais**
**9.** Vous vous êtes senti(e) calme et détendu(e) ?	☐	☐	☐	☐	☐	☐
**10.** Vous vous êtes senti(e) débordant(e) d'énergie ?	☐	☐	☐	☐	☐	☐

11. Vous vous êtes senti(e)
triste et abattu(e) ? ☐ ☐ ☐ ☐ ☐ ☐

12. Au cours de ces 4 dernières semaines y a t-il eu des moments où votre état de santé phvsique ou émotionnel, vous a gêné(e) dans votre vie sociale et vos relations avec les autres, votre famille, vos amis, vos connaissances?

(Cochez une seule réponse)

- En permanence...☐
- Une bonne partie du temps ....☐
- De temps en temps ....☐
- Rarement ....☐
- Jamais ....☐

Gandek B, Ware JE, Aaronson NK, Apolone G, Bjorner JB, Brazier JE, Bullinger M, Kaasa S, Leplege A, Prieto L, Sullivan M. Cross-validation of item selection and scoring for the SF-12 Health Survey in nine countries: results from the IQOLA Project. International Quality of Life Assessment. J Clin Epidemiol. 1998 Nov;51(11):1171-8.

SF-12 Health Survey

This survey asks for your views about your health. This information will help keep track of how you feel and how well you are able to do your usual activities. **Answer each question by choosing just one answer**. If you are unsure how to answer a question, please give the best answer you can.

1. **In general, would you say your health is**:

☐1 Excellent ☐2 Very good ☐3 Good ☐4 Fair ☐5 Poor

The following questions are about activities you might do during a typical day. Does your health now limit you in these activities? If so, how much?

	**YES, limited a lot**	**YES, limited a little**	**NO, not limited at all**
2. **Moderate activities** such as moving a table, pushing a vacuum cleaner, bowling, or playing golf.	☐1	☐2	☐3
3. Climbing **several** flights of stairs.	☐1	☐2	☐3

During the past 4 weeks, have you had any of the following problems with your work or other regular daily activities as a result of your physical health?

	**YES**	**NO**
4. **Accomplished less** than you would like.	☐1	☐2
5. Were limited in the **kind** of work or other activities.	☐1	☐2

During the past 4 weeks, have you had any of the following problems with your work or other regular daily activities as a result of any emotional problems (such as feeling depressed or anxious)?

	**YES**	**NO**
6. **Accomplished less** than you would like.	☐1	☐2
7. Did work or activities **less carefully than usual**.	☐1	☐2

8. **During the past 4 weeks, how much did pain interfere with your normal work (including work outside the home and housework)?**

☐1 Not at all ☐2 A little bit ☐3 Moderately ☐4 Quite a bit ☐5 Extremely

These questions are about how you have been feeling during the past 4 weeks.
For each question, please give the one answer that comes closest to the way you have been feeling.

How much of the time during the past 4 weeks...

	All of the time	Most of the time	A good bit of the time	Some of the time	A little of the time	None of the time
9. Have you felt calm & peaceful?	☐1	☐2	☐3	☐4	☐5	☐6
10. Did you have a lot of energy?	☐1	☐2	☐3	☐4	☐5	☐6
11. Have you felt down-hearted and blue?	☐1	☐2	☐3	☐4	☐5	☐6

12. **During the past 4 weeks, how much of the time has your physical health or emotional problems interfered with your social activities (like visiting friends, relatives, etc.)?**

☐1 All of the time ☐2 Most of the time ☐3 Some of the time ☐4 A little of the time ☐5 None of the time

APPENDIX B

QUESTIONNAIRE DE CROYANCES ET ATTENTES

Grâce au questionnaire ci-dessous, nous cherchons à savoir si, en ce moment, vous croyez que le traitement que vous suivez va aider à réduire vos symptômes.

Entourez le chiffre qui correspond le mieux à votre réponse.

En ce moment, dans quelle mesure le traitement que l'on vous a proposé vous semble-t-il logique ?

Pas du tout logique								Tout à fait logique
☐	☐	☐	☐	☐	☐	☐	☐	☐
1	2	3	4	5	6	7	8	9

En ce moment, dans quelle mesure pensez-vous que ce traitement sera utile pour réduire vos symptômes ?

Pas du tout utile								Tout à fait utile
☐	☐	☐	☐	☐	☐	☐	☐	☐
1	2	3	4	5	6	7	8	9

Recommandez-vous le traitement à un ami qui a des problèmes semblables ?

Pas du tout								Tout à fait
☐	☐	☐	☐	☐	☐	☐	☐	☐
1	2	3	4	5	6	7	8	9

À la fin de la période de traitement, quelle amélioration de vos symptômes pensez-vous obtenir ?

Aucune amélioration								Amélioration complète
☐	☐	☐	☐	☐	☐	☐	☐	☐
1	2	3	4	5	6	7	8	9

En ce moment, dans quelle mesure avez-vous la sensation que ce traitement sera utile pour réduire vos symptômes ?

Pas du tout utile								Tout à fait utile
☐	☐	☐	☐	☐	☐	☐	☐	☐
1	2	3	4	5	6	7	8	9

Borkovec T D and Nau S D. Credibility of analogue therapy rationales. Journal of Behavior Therapy and Experimental Psychiatry. 1972:3;257-260. Coste J, Tarquinio, Rouquette A, Montelac S, Pouchot J, Adaptation transculturelle et validation de la version française du questionnaire Croyances et Attentes. Exploration complémentaire des concepts mesurés et de leurs relations, Psychologie française.2019:65(2)

Therapy Evaluation Form

We would like you to indicate below how much you believe, right now, that the therapy you are receiving will help to improve your lifestyle / functioning. Belief usually has two aspects to it: (1) what one thinks will happen and (2) what one feels will happen. Sometimes these are similar; sometimes they are different. Please answer the questions below. In the first set, answer in terms of what you think. In the second set answer in terms of what you really and truly feel. We do not want your course convenors to ever see these ratings, so please keep the sheet covered when you are done.

Set I

1. At this point, how logical does the course offered to you seem?

1	2	3	4	5	6	7	8	9
not at all logical				somewhat logical				very logical

2. At this point, how successfully do you think this course will be in raising the quality of your functioning?

1	2	3	4	5	6	7	8	9
not at all useful				somewhat useful				very useful

3. How confident would you be in recommending this course to a friend who experiences similar problems?

1	2	3	4	5	6	7	8	9
none at all confident				Somewhat confident				very confident

4. By the end of the course, how much improvement in your functioning do you think will occur?

0% 10% 20% 30% 40% 50% 60% 70% 80% 90% 100%

Set II

For this set, close your eyes for a few moments, and try to identify what you really feel about the course and its likely success. Then answer the following questions.

1. At this point, how much do you really feel that the course will help you to improve your functioning?

1	2	3	4	5	6	7	8	9
not at all				somewhat				very much

2. By the end of the course, how much improvement in your functioning do you really feel will occur?

0% 10% 20% 30% 40% 50% 60% 70% 80% 90% 100%

APPENDIX C

Questionnaire de Roland Morris

Lorsque vous avez mal au dos, vous pouvez avoir du mal à réaliser certaines des activités que vous faites d'habitude.

Vous trouverez ci-dessous une liste de phrases qui ont été utilisées par des personnes souffrant de mal de dos pour décrire leur situation. A la lecture de ces phrases, certaines se détacheront peut-être car elles décrivent votre situation *aujourd'hui*. En lisant cette liste, pensez à votre situation *aujourd'hui*. Quand vous lirez une phrase qui décrit votre situation *aujourd'hui*, cochez la case qui se trouve à côté de cette phrase. Si la phrase ne vous correspond pas, laissez un blanc et passez à la phrase suivante. **Faites bien attention à ne cocher que les phrases qui décrivent votre situation *aujourd'hui*.**

1. A cause de mon mal de dos, je reste pratiquement toute la journée à la maison. □
2. Je change souvent de position pour essayer de soulager mon mal de dos. □
3. A cause de mon mal de dos, je marche plus lentement que d'habitude. □
4. A cause de mon mal de dos, je ne fais aucune des tâches que je fais d'habitude à la maison. □
5. A cause de mon mal de dos, je m'aide de la rampe pour monter les escaliers. □
6. A cause de mon mal de dos, je m'allonge plus souvent que d'habitude pour me reposer. □
7. A cause de mon mal de dos, j'ai besoin de m'agripper à quelque chose pour me lever d'un fauteuil □
8. A cause de mon mal de dos, je demande aux autres de faire certaines choses à ma place. □
9. A cause de mon mal de dos, je m'habille plus lentement que d'habitude. □
10. A cause de mon mal de dos, je ne peux rester debout que de courts instants. □
11. A cause de mon mal de dos, j'évite de me pencher ou de m'agenouiller. □
12. A cause de mon mal de dos, j'ai du mal à me lever d'une chaise. □
13. J'ai pratiquement tout le temps mal au dos. □
14. A cause de mon mal de dos, j'ai du mal à me retourner dans mon lit. □
15. A cause de mon mal de dos, je n'ai pas beaucoup d'appétit. □
16. A cause de mon mal de dos, j'ai du mal à enfiler mes chaussettes (ou mes bas, ou mes collants). □
17. A cause de mon mal de dos, je ne peux marcher que sur de courtes distances. □
18. A cause de mon mal de dos, je dors moins que d'habitude. □
19. A cause de mon mal de dos, j'ai besoin de l'aide de quelqu'un pour m'habiller. □
20. A cause de mon mal de dos, je reste assis(e) pratiquement toute la journée. □
21. A cause de mon mal de dos, j'évite les tâches pénibles à la maison. □
22. A cause de mon mal de dos, je suis plus irritable et de plus mauvaise humeur avec les autres que d'habitude. □
23. A cause de mon mal de dos, je monte les escaliers plus lentement que d'habitude. □
24. A cause de mon mal de dos, je reste pratiquement toute la journée au lit.

Zerkak D, Métivier JC, Fouquet B, Beaudreuil J. Validation of a French version of Roland-Morris questionnaire in chronic low back pain patients. Ann Phys Rehabil Med. 2013 Dec;56(9-10):613-20. doi: 10.1016/j.rehab.2013.08.006.

Roland-Morris Low Back Pain and Disability Questionnaire (RMQ)

Instructions

Patient name:________________________ File #:__________ Date:__________

Please read instructions: When your back hurts, you may find it difficult to do some of the things you normally do. Mark only the sentences that describe you today.

- ☐ I stay at home most of the time because of my back.
- ☐ I change position frequently to try to get my back comfortable.
- ☐ I walk more slowly than usual because of my back.
- ☐ Because of my back, I am not doing any jobs that I usually do around the house.
- ☐ Because of my back, I use a handrail to get upstairs.
- ☐ Because of my back, I lie down to rest more often.
- ☐ Because of my back, I have to hold on to something to get out of an easy chair.
- ☐ Because of my back, I try to get other people to do things for me.
- ☐ I get dressed more slowly than usual because of my back.
- ☐ I only stand up for short periods of time because of my back.
- ☐ Because of my back, I try not to bend or kneel down.
- ☐ I find it difficult to get out of a chair because of my back.
- ☐ My back is painful almost all of the time.
- ☐ I find it difficult to turn over in bed because of my back.
- ☐ My appetite is not very good because of my back.
- ☐ I have trouble putting on my socks (or stockings) because of the pain in my back.
- ☐ I can only walk short distances because of my back pain.
- ☐ I sleep less well because of my back.
- ☐ Because of my back pain, I get dressed with the help of someone else.
- ☐ I sit down for most of the day because of my back.
- ☐ I avoid heavy jobs around the house because of my back.
- ☐ Because of back pain, I am more irritable and bad tempered with people than usual.
- ☐ Because of my back, I go upstairs more slowly than usual.
- ☐ I stay in bed most of the time because of my back.

APPENDIX D

Score de rumination

Pour chacune des affirmations suivantes, veuillez exprimer le degré d'accord ou de désaccord en utilisant le barème suivant : pas du tout d'accord (1), pas d'accord (2), ni ne désaccord, ni d'accord (3), d'accord (4), tout à fait d'accord (5). Cochez la case correspondante.

Questionnaire de rumination/réflexion	pas du tout d'accord	pas d'accord	ni en désaccord, ni en accord	d'accord	tout à fait d'accord
	1	2	3	4	5
**Rumination**					
1. Je me focalise souvent sur des aspects de ma personne auxquels j'aimerais cesser de penser					
2. J'ai toujours l'impression de ressasser des choses que j'ai récemment dites ou faites					
3. Il m'est parfois difficile de faire cesser les pensées centrées sur moi-même					
4. Longtemps après un désaccord ou une dispute, mes pensées restent focalisées sur ce qui s'est passé					
5. J'ai tendance à ruminer ou à rester fixé(e) sur des choses, longtemps après qu'elles soient arrivées					
6. *Je ne perds pas mon temps à repenser à des choses qui sont passées et terminées*					
7. Souvent, je rejoue dans mon esprit la manière dont j'ai agi dans telle situation passée					
8. Je me retrouve souvent en train de reconsidérer quelque chose que j'ai fait					
9. *Je ne rumine ou ne reste jamais longtemps fixé sur moi-même*					
10. *Il m'est facile de me sortir de l'esprit des pensées indésirables*					
11. Je réfléchis souvent à des épisodes de ma vie dont je ne devrais plus me préoccuper					
12. Je passe beaucoup de temps à repenser à des moments où j'ai été gêné, déçu					
**Réflexion**					
13. *Les pensées philosophiques ou abstraites ne m'intéressent pas beaucoup*					
14. *Je ne suis pas vraiment du genre méditatif*					
15. J'adore explorer mon moi intérieur					
16. Je suis fasciné par mon point de vue et mes sentiments sur les choses					
17. *Les pensées introspectives ou réflexives ne m'intéressent pas trop*					
18. J'aime beaucoup analyser pourquoi je fais les choses					
19. Les gens disent souvent je que je suis une personnalité profonde et introspective					
20. *Je n'aime pas trop l'auto-analyse*					
21. Par nature, je suis curieux de moi-même					
22. J'aime beaucoup méditer sur la nature et le sens des choses					

23. Souvent, j'aime envisager ma vie sous un angle philosophique					
24. *Ça ne m'amuse pas de me contempler*					

Trapnell PD, Campbell JD. Private self-consciousness and the five-factor model of personality: distinguishing rumination from reflection. J Pers Soc Psychol. 1999;76(2):284-304.
Translation by A. Roren and C. Nguyen, Back-translation by J. Robertson (translator)

RRQ

Instructions:

For each of the statements located on the next two pages, please indicate your level of agreement or disagreement by circling one of the scale categories to the right of each statement. Use the scale as shown below:

Strongly Disagree 1	Disagree 2	Neutral 3	Agree 4	Strongly Agree 5

1.	My attention is often focused on aspects of myself I wish I'd stop thinking about................................	1	2	3	4	5
2.	I always seem to be "re-hashing" in my mind recent things I've said or done................................	1	2	3	4	5
3.	Sometimes it is hard for me to shut off thoughts about myself................................	1	2	3	4	5
4.	Long after an argument or disagreement is over with, my thoughts keep going back to what happened.	1	2	3	4	5
5.	I tend to "ruminate" or dwell over things that happen to me for a really long time afterward.	1	2	3	4	5
6.	I don't waste time re-thinking things that are over and done with................................	1	2	3	4	5
7.	Often I'm playing back over in my mind how I acted in a past situation.	1	2	3	4	5
8.	I often find myself re-evaluating something I've done................................	1	2	3	4	5
9.	I never ruminate or dwell on myself for very long................................	1	2	3	4	5
10.	It is easy for me to put unwanted thoughts out of my mind.	1	2	3	4	5
11.	I often reflect on episodes in my life that I should no longer concern myself with................................	1	2	3	4	5
12.	I spend a great deal of time thinking back over my embarrassing or disappointing moments.	1	2	3	4	5

Strongly Disagree	Disagree	Neutral	Agree	Strongly Agree
1	2	3	4	5

13.	Philosophical or abstract thinking doesn't appeal to me that much.	1	2	3	4	5
14.	I'm not really a meditative type of person.	1	2	3	4	5
15.	I love exploring my "inner" self.	1	2	3	4	5
16.	My attitudes and feelings about things fascinate me.	1	2	3	4	5
17.	I don't really care for introspective or self-reflective thinking.	1	2	3	4	5
18.	I love analyzing why I do things.	1	2	3	4	5
19.	People often say I'm a "deep", introspective type of person.	1	2	3	4	5
20.	I don't care much for self-analysis.	1	2	3	4	5
21.	I'm very self-inquisitive by nature.	1	2	3	4	5
22.	I love to meditate on the nature and meaning of things.	1	2	3	4	5
23.	I often love to look at my life in philosophical ways.	1	2	3	4	5
24.	Contemplating myself isn't my idea of fun.	1	2	3	4	5

APPENDIX E

Questionnaire de strategies de coping (CSQ)

Indiquez, pour chacune des stratégies suivantes, si vous l'avez utilisée pour faire face à votre problème en sachant que : 1 = non 2 = plutôt non 3 = plutôt oui 4 = oui

	1	2	3	4
1. J'ai essayé de prendre de la distance par rapport à la douleur, comme si elle était dans le corps de quelqu'un d'autre				
2. J'ai essayé de penser à quelque chose d'agréable				
3. J'ai trouvé que c'était terrible et j'ai eu l'impression que ça n'irait jamais mieux				
4. J'ai parlé à quelqu'un de ce que je ressentais				
5. J'ai sollicité l'aide d'un professionnel et j'ai fait ce qu'on m'a conseillé				
6. J'ai trouvé que c'était affreux et j'ai eu l'impression que la douleur m'écrasait				
7. J'ai demandé des conseils à une personne digne de respect et je les ai suivis				
8. J'ai prié Dieu ou le destin pour que ma douleur ne dure pas				
9. J'ai discuté avec quelqu'un pour en savoir plus au sujet de ma douleur				
10. J'ai essayé de penser à la douleur comme si elle était séparée de mon corps				
11. J'ai contenu (gardé pour moi) mes émotions				
12. Je n'ai pas prêté attention à la douleur				
13. J'ai parlé avec quelqu'un qui pouvait agir concrètement au sujet de ma douleur				
14. J'ai fait comme si je ne souffrais pas				
15. J'ai eu peur que la douleur ne cesse pas				
16. J'ai repensé à des moments agréables du passé				
17. J'ai essayé de ne pas m'isoler				
18. J'ai pensé à des personnes avec lesquelles j'aime faire des choses				
19. J'ai prié pour que la douleur disparaisse				
20. J'ai imaginé que la douleur était en dehors de mon corps				
21. J'ai accepté la sympathie et la compréhension de quelqu'un				

22. Bien que j'ai eu mal, j'ai continué mes activités				
23. J'ai eu l'impression que je ne pouvais plus supporter la douleur				
24. J'ai recherché la compagnie des autres, j'ai essayé de ne pas rester seul(e)				
25. J'ai ignoré la douleur				
26. J'ai compté sur ma foi en Dieu ou dans le destin				
27. J'ai eu l'impression de ne plus pouvoir aller de l'avant				
28. J'ai pensé à des choses que j'aime faire				
29. J'ai fait comme si la douleur ne faisait pas partie de moi				

Rosenstiel, AK and Keefe FJ. The Use of Coping Strategies in Chronic Low Back Pain Patients: Relationship to Patient Characteristics and Current Adjustment. Pain. 1983;17: 33-44.
Irachabal S, Koleck M, Rascle N, Bruchon-Schweitzer M. [Pain coping strategies: French adaptation of the coping strategies questionnaire (CSQ-F)]. Encephale. 2008 Jan;34(1):47-53. doi: 10.1016/j.encep.2006.11.002.

APPENDIX F

Questionnaire FABQ

Voici des notions que d'autres patients nous ont exprimées au sujet de leur douleur. Pour chaque affirmation veuillez entourer un chiffre de 0 à 6 pour indiquer combien les activités physiques telles se pencher, soulever, marcher, conduire, influent ou pourraient influer sur votre mal de dos. Entre 0 et 6 le chiffre que vous entourerez exprimera le degré d'accord ou de désaccord avec la proposition.

	Désaccord total			Incertain			Accord total
1. Ma douleur a été provoquée par l'activité physique	0	1	2	3	4	5	6
2. L'activité physique aggrave ma douleur	0	1	2	3	4	5	6
3. L'activité physique pourrait abîmer mon dos	0	1	2	3	4	5	6
4. Je ne devrais pas faire d'activités physiques lesquelles pourraient aggrave ma douleur	0	1	2	3	4	5	6
5. Je ne peux pas faire d'activités physiques lesquelles pourraient aggraver m douleur	0	1	2	3	4	5	6

Les affirmations suivantes concernent la manière dont votre travail habituel influe ou pourrait influer sur votre mal de dos.

	Désaccord total			Incertain			Accord total
6. La douleur a été provoquée par mon travail ou par un accident de travail	0	1	2	3	4	5	6
7. Mon travail a aggravé ma douleur	0	1	2	3	4	5	6
8. J'ai droit à une indemnisation pour ma douleur	0	1	2	3	4	5	6
9. Mon travail est trop dur pour moi	0	1	2	3	4	5	6
10. Mon travail augmente ou pourrait augmenter mes douleurs	0	1	2	3	4	5	6
11. Mon travail pourrait abîmer mon dos	0	1	2	3	4	5	6
12. Je ne devrais pas faire mon travail habituel avec ma douleur actuelle	0	1	2	3	4	5	6
13. Je ne peux pas faire mon travail habituel avec ma douleur actuelle	0	1	2	3	4	5	6
14. Je ne peux pas faire mon travail habituel avant que mes douleurs soien traitées efficacement	0	1	2	3	4	5	6
15. Je ne pense pas que je pourrai reprendre mon travail habituel avant 3 mois	0	1	2	3	4	5	6
16. Je ne pense pas que je pourrai un jour être capable de reprendre ce travail	0	1	2	3	4	5	6

Chaory K, Fayad F, Rannou F, Lefèvre-Colau MM, Fermanian J, Revel M, Poiraudeau S. Validation of the French version of the fear avoidance belief questionnaire. Spine (Phila Pa 1976). 2004 ;15;29(8):908-13.

Here are some of the things which other patients have told us about their pain. For each statement please circle any number from 0 to 6 to say how much physical activities such as bending, lifting, walking or driving affect or would affect your back pain.

	COMPLETELY DISAGREE			UNSURE			COMPLETELY AGREE
1. My pain was caused by physical activity	0	1	2	3	4	5	6
2. Physical activity makes my pain worse	0	1	2	3	4	5	6
3. Physical activity might harm my back	0	1	2	3	4	5	6
4. I should not do physical activities which (might) make my pain worse	0	1	2	3	4	5	6
5. I cannot do physical activities which (might) make my pain worse	0	1	2	3	4	5	6

The following statements are about how your normal work affects or would affect your back pain.

	COMPLETELY DISAGREE			UNSURE			COMPLETELY AGREE
6. My pain was caused by my work or by an accident at work	0	1	2	3	4	5	6
7. My work aggravated my pain	0	1	2	3	4	5	6
8. I have a claim for compensation for my pain	0	1	2	3	4	5	6
9. My work is too heavy for me	0	1	2	3	4	5	6
10. My work makes or would make my pain worse	0	1	2	3	4	5	6
11. My work might harm my back	0	1	2	3	4	5	6
12. I should not do my normal work with my present pain	0	1	2	3	4	5	6
13. I cannot do my normal work with my present pain	0	1	2	3	4	5	6
14. I cannot do my normal work until my pain is treated	0	1	2	3	4	5	6
15. I do not think that I will be back to my normal work within 3 months	0	1	2	3	4	5	6
16. I do not think that I will ever be able to go back to that work	0	1	2	3	4	5	6

APPENDIX G

Questionnaire HAD

Les médecins savent que les émotions jouent un rôle important dans la plupart des maladies. Si votre médecin est au courant des émotions que vous éprouvez, il pourra mieux vous aider. Ce questionnaire a été conçu de façon à permettre à votre médecin de se familiariser avec ce que vous éprouvez vous-même sur le plan émotif.
- Ne faites pas attention aux lettres imprimées en haut des colonnes à la gauche du questionnaire.
- Lisez chaque série de questions et soulignez la réponse qui exprime le mieux ce que vous avez éprouvé au cours de la semaine qui vient de s'écouler.
- Ne vous attardez pas sur la réponse à faire ; votre réaction immédiate à chaque question fournira probablement une meilleure indication de ce que vous éprouvez qu'une réponse longuement méditée.

D	A	
		**JE ME SENS TENDU OU ÉNERVÉ** :
	3	- La plupart du temps
	2	- Souvent
	1	- De temps en temps
	0	- Jamais
		**JE PRENDS PLAISIR AUX MÊMES CHOSES QU'AUTREFOIS** :
0		- Oui, tout autant
1		- Pas autant
2		- Un peu seulement
3		- Presque plus
		**J'AI UNE SENSATION DE PEUR COMME SI QUELQUE CHOSE D'HORRIBLE ALLAIT M'ARRIVER** :
	3	- Oui, très nettement
	2	- Oui, mais ce n'est pas grave
	1	- Un peu, mais cela ne m'inquiète pas
	0	- Plus du tout
		**JE RIS FACILEMENT ET VOIS LE BON CÔTÉ DES CHOSES** :
0		- Autant que par le passé
1		- Plus autant qu'avant
2		- Vraiment moins qu'avant
3		- Plus du tout
		**JE ME FAIS DU SOUCI** :
	3	- Très souvent
	2	- Assez souvent
	1	- Occasionnellement
	0	- Très occasionnellement
		**JE SUIS DE BONNE HUMEUR** :
3		- Jamais
2		- Rarement
1		- Assez souvent
0		- La plupart du temps

D	A	
		**JE PEUX RESTER TRANQUILLEMENT ASSIS À NE RIEN FAIRE ET ME SENTIR DECONTRACTÉ :**
	0	- Oui, quoi qu'il arrive
	1	- Oui, en général
	2	- Rarement
	3	- Jamais
		**J'AI L'IMPRESSION DE FONCTIONNER AU RALENTI** :
3		- Presque toujours
2		- Très souvent
1		- Parfois
0		- Jamais
		**J'ÉPROUVE DES SENSATIONS DE PEUR ET J'AI L'ESTOMAC NOUÉ** :
	0	- Jamais
	1	- Parfois
	2	- Assez souvent
	3	- Très souvent
		**JE NE M'INTÉRESSE PLUS À MON APPARENCE** :
3		- Plus du tout
2		- Je n'y accorde pas autant d'attention que je le devrais
1		- Il se peut que je n'y fasse plus autant attention
0		- J'y prête autant d'attention que par le passé
		**J'AI LA BOUGEOTTE ET N'ARRIVE PAS À TENIR EN PLACE** :
	3	- Oui, c'est tout-à-fait le cas
	2	- Un peu
	1	- Pas tellement
	0	- Pas du tout
		**JE ME RÉJOUIS D'AVANCE À L'IDÉE DE FAIRE CERTAINES CHOSES** :
0		- Autant qu'avant
1		- Un peu moins qu'avant
2		- Bien moins qu'avant
3		- Presque jamais
		**J'ÉPROUVE DES SENSATIONS SOUDAINES DE PANIQUES** :
	3	- Vraiment très souvent
	2	- Assez souvent
	1	- Pas souvent ou parfois
	0	- Jamais
		**JE PEUX PRENDRE PLAISIR À UN BON LIVRE OU À UNE BONNE ÉMISSION DE RADIO OU DE TÉLÉVISION** :
0		- Souvent
1		- Parfois
2		- Rarement
3		- Très rarement

Hospital Anxiety and Depression Scale (HADS)

Tick the box beside the reply that is closest to how you have been feeling in the past week.
Don't take too long over you replies: your immediate is best.

D	A		D	A	
		**I feel tense or 'wound up':**			**I feel as if I am slowed down:**
	3	Most of the time	3		Nearly all the time
	2	A lot of the time	2		Very often
	1	From time to time, occasionally	1		Sometimes
	0	Not at all	0		Not at all
		**I still enjoy the things I used to enjoy:**			**I get a sort of frightened feeling like 'butterflies' in the stomach:**
0		Definitely as much		0	Not at all
1		Not quite so much		1	Occasionally
2		Only a little		2	Quite Often
3		Hardly at all		3	Very Often
		**I get a sort of frightened feeling as if something awful is about to happen:**			**I have lost interest in my appearance:**
	3	Very definitely and quite badly	3		Definitely
	2	Yes, but not too badly	2		I don't take as much care as I should
	1	A little, but it doesn't worry me	1		I may not take quite as much care
	0	Not at all	0		I take just as much care as ever
		**I can laugh and see the funny side of things:**			**I feel restless as I have to be on the move:**
0		As much as I always could		3	Very much indeed
1		Not quite so much now		2	Quite a lot
2		Definitely not so much now		1	Not very much
3		Not at all		0	Not at all
		**Worrying thoughts go through my mind:**			**I look forward with enjoyment to things:**
	3	A great deal of the time	0		As much as I ever did
	2	A lot of the time	1		Rather less than I used to
	1	From time to time, but not too often	2		Definitely less than I used to
	0	Only occasionally	3		Hardly at all
		**I feel cheerful:**			**I get sudden feelings of panic:**
3		Not at all		3	Very often indeed
2		Not often		2	Quite often
1		Sometimes		1	Not very often
0		Most of the time		0	Not at all
		**I can sit at ease and feel relaxed:**			**I can enjoy a good book or radio or TV program:**
	0	Definitely	0		Often
	1	Usually	1		Sometimes
	2	Not Often	2		Not often
	3	Not at all	3		Very seldom

Please check you have answered all the questions

APPENDIX H

Semi-open patient questionnaire

Satisfaction / activity: gardening (horticultural therapy) / handiwork: on a scale from 0 to 100, (0 representing total absence of satisfaction and 100 total satisfaction), rate your satisfaction with the gardening (horticultural therapy) / handiwork activity.

0 —————————————————— 100

Acceptability: on a scale from 0 to 100 (0: not acceptable, 100: totally acceptable), rate your experience of gardening (horticultural therapy) / handiwork.

0 —————————————————— 100

In the future, would you like to benefit again from a rehabilitation program that includes gardening (horticultural therapy) / handiwork?

- ☐ Yes
- ☐ No

Briefly explain why

APPENDIX I

Semi-open questionnaire for caregivers

Acceptability: on a scale from 0 to 100 (0: not acceptable; 100: totally acceptable), rate your experience of gardening (horticultural therapy) / handiwork activities with chronic low back pain patients:

0 ———————————————————————— 100

In the future, would you like to use gardening (horticultural therapy) / handiwork again for the rehabilitation of chronic low back pain patients?

- ☐ Yes
- ☐ No

Briefly explain why:

APPENDIX J

Poster for patient recruitment:

__16 patients__ doivent être inclus dans cette recherche

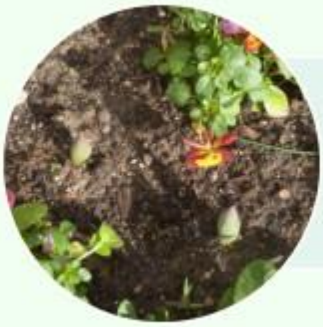

Etude HORTICARE

- ✓ Vous avez une lombalgie chronique (épisode actuel d'une durée > 3 mois),
- ✓ La rééducation est indiquée dans la prise en charge de votre lombalgie,
- ✓ Vous occupez un emploi rémunéré,
- ✓ Vous n'avez pas de contre-indication à l'IRM.

- ✓ Participez à l'étude HORTICARE sur l'effet de l'horticulture thérapeutique sur le cerveau (zone du cerveau traitant les informations de douleur et émotions associées aux douleurs) chez les personnes lombalgiques chroniques.

*Service de Rééducation et de Réadaptation de l'Appareil Locomoteur et des Pathologies du Rachis, **Hôpital COCHIN***

APPENDIX K

Patient recruitment brochure :

ETUDE HORTICARE

Objectif de l'étude: Evaluer l'effet de l'horticulture thérapeutique sur le cerveau (zone du cerveau traitant les informations de douleur et émotions associées aux douleurs), chez les personnes lombalgiques chroniques.

Organisation pratique:

- Pour participer à cette étude, vous devez être lombalgique chronique, ne pas avoir de contre-indications à l'IRM, occuper un emploi rémunéré et ne pas avoir eu d'arrêt de travail de plus de 3 mois au cours de la dernière année.

- Si vous acceptez de participer à cette étude, vous aurez : 2 ateliers de jardinage ou de bricolage puis 1 semaine de pause, puis 2 ateliers de jardinage ou de bricolage. Ces ateliers d'une durée de 90 minutes ont lieu dans notre service et sont supervisés par un ergothérapeute. Pour le jardinage, nous disposons d'une serre.

Service de Rééducation et de Réadaptation de l'Appareil Locomoteur et des Pathologies du Rachis

- Vous aurez 3 IRMs du cerveau au total au cours de votre participation (une le jour de l'inclusion, une à la fin de la 1ère activité et à la dernière à la fin de la 2ème activité (fin de l'intervention). Ces IRM seront réalisées à l'Hôpital Sainte-Anne, dans le service de radiologie du Pr Catherine Oppenheim.

Chaque IRM dure 35 minutes environ. L'IRM est un examen non irradiant qui a de rares contre-indications : présence de matériel métallique intracorporel, d'un stimulateur cardiaque ou cérébral, grossesse en cours, claustrophobie incontrôlable...

Les ateliers et les IRMs ont lieu le matin.

- Pour cette recherche, aucun autre examen médical n'est prévu en dehors des IRM. On vous demandera néanmoins de compléter différents questionnaires portant sur votre qualité de vie, vos limitations fonctionnelles, vos croyances, votre ressenti et vos réactions, en lien avec la lombalgie.

Pour toute information complémentaire, vous pouvez contacter: Alexandra ROREN, alexandra.roren@aphp.fr, 0158411371.

Service de Rééducation et de Réadaptation de l'Appareil Locomoteur et des Pathologies du Rachis
