# Supplementary figures and images for: Effects of horticultural therapy versus handiwork on anterior cingulate cortex activity in people with chronic low back pain: A randomized, controlled, cross-over, pilot study
Source: PLoS One. 2024 Dec 17;19(12):e0313920. doi: 10.1371/journal.pone.0313920 (PMC11651560; doi:10.1371/journal.pone.0313920)

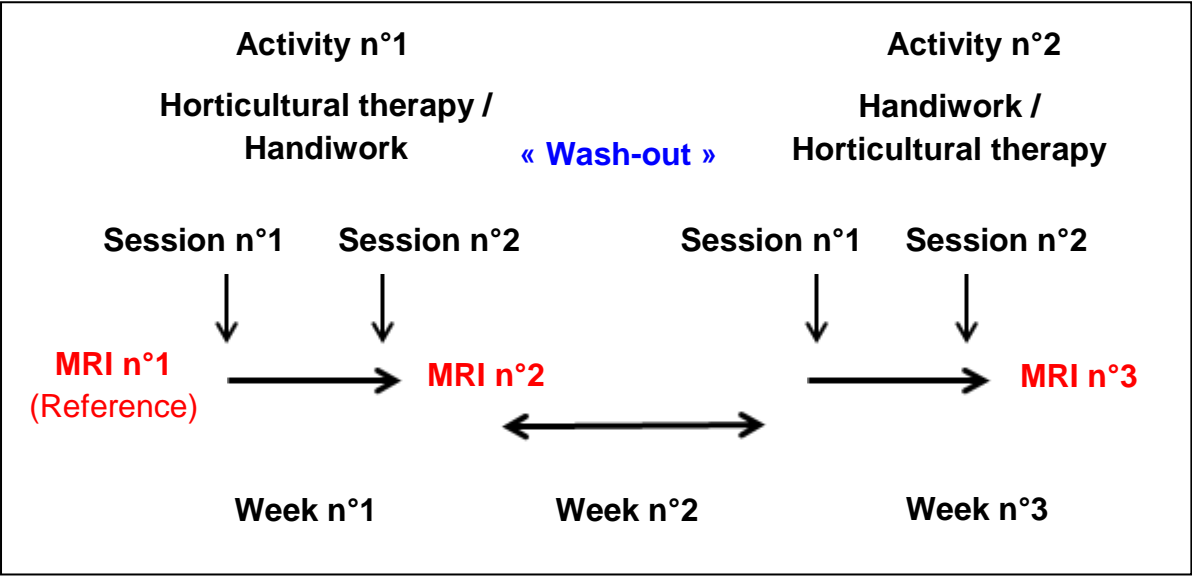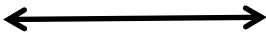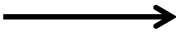

Supplement: S3 Fig — (PDF) [file pone.0313920.s004.pdf]

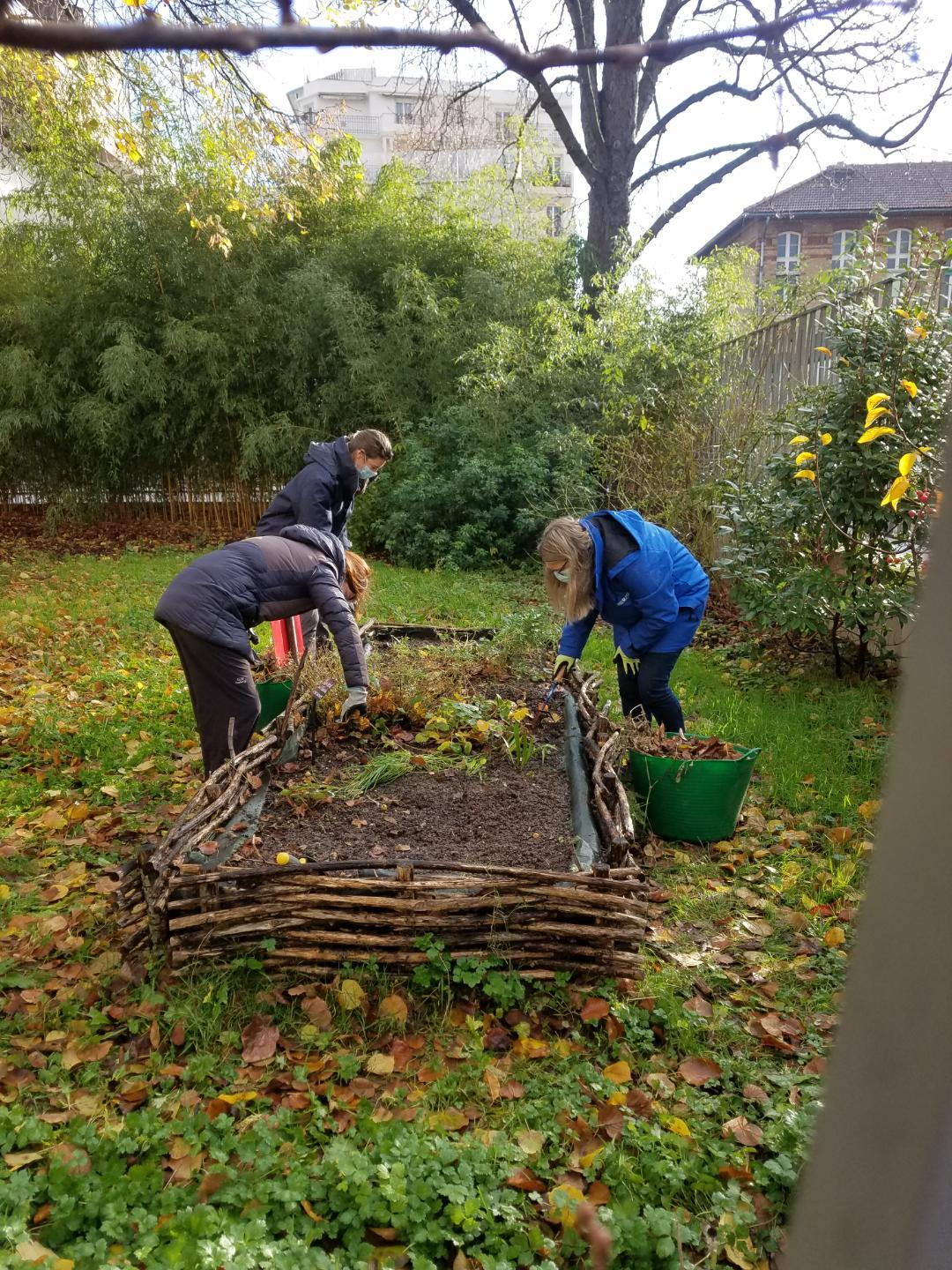

Supplement: S4 Fig — (TIF) [file pone.0313920.s005.tif]

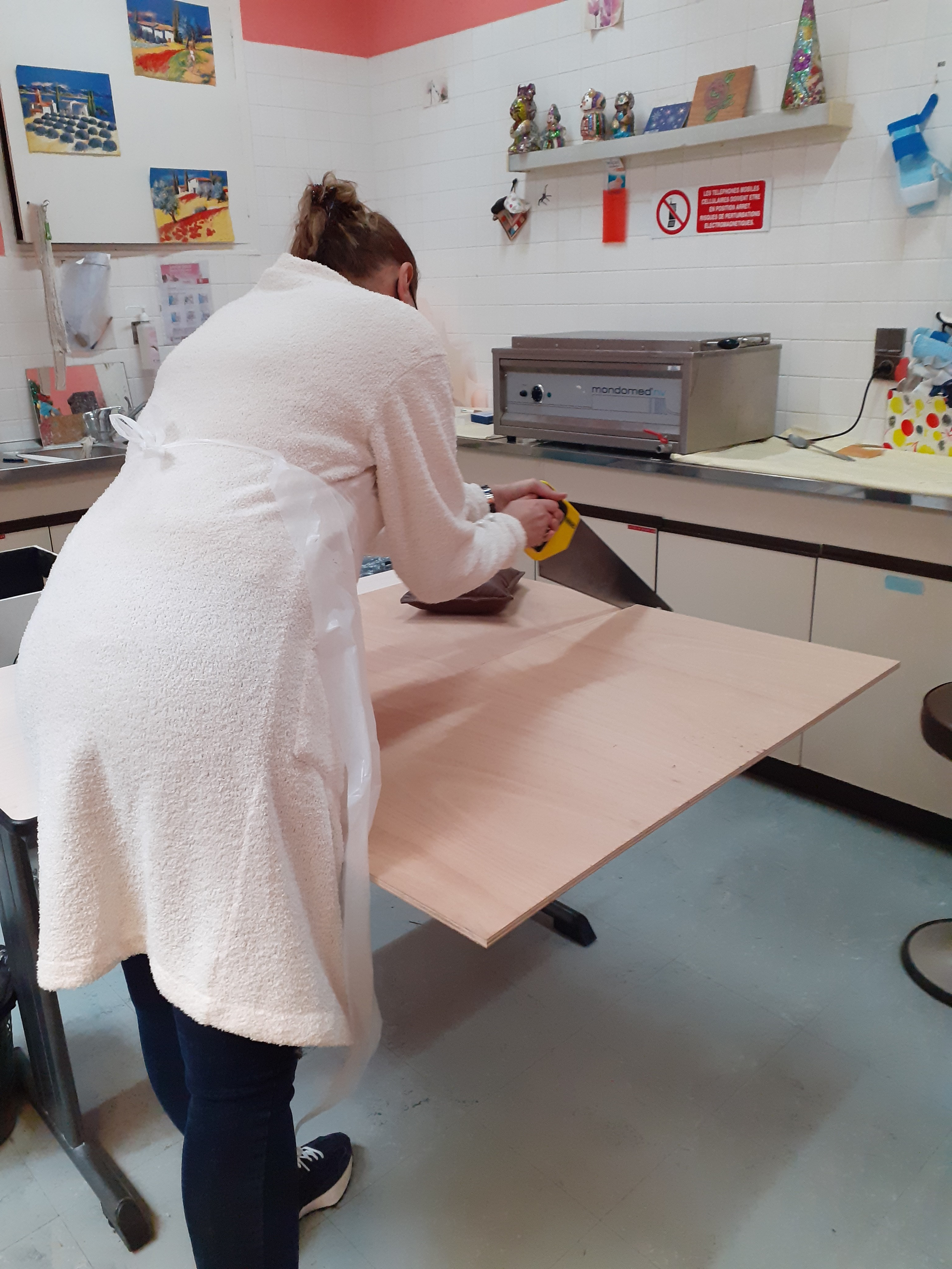

Supplement: S5 Fig — (TIF) [file pone.0313920.s006.tif]

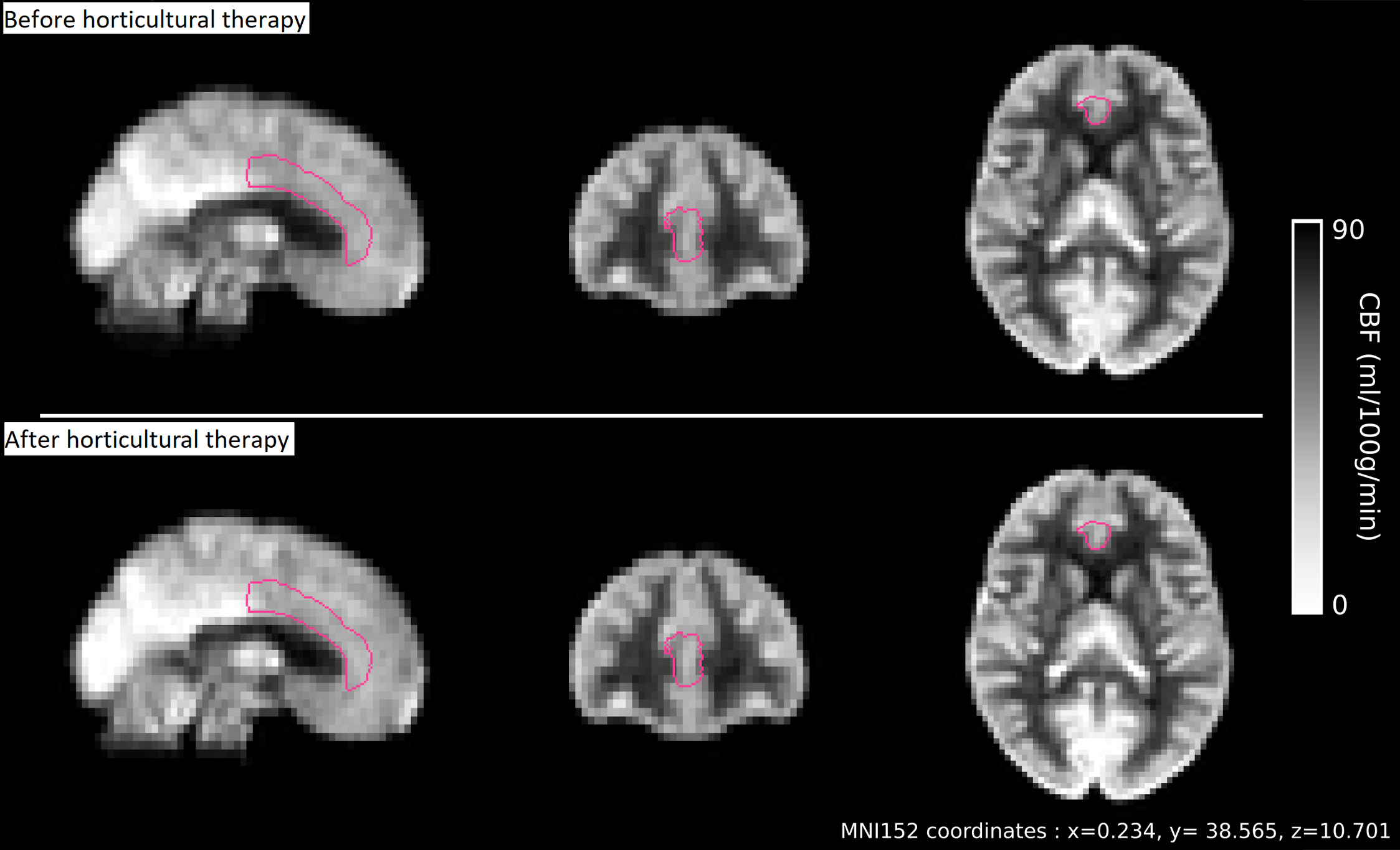

Supplement: S6 Fig — The pink outline corresponds to the anterior division of the cingulate gyrus of the Harvard-Oxford cortical structural atlases with a threshold at the probability of 50%. The quality of the image is linked to the low intrinsic resolution of the ASL. (TIF) [file pone.0313920.s007.tif]
