# Supplementary material for: Effects of horticultural therapy versus handiwork on anterior cingulate cortex activity in people with chronic low back pain: A randomized, controlled, cross-over, pilot study
Source: PLoS One. 2024 Dec 17;19(12):e0313920. doi: 10.1371/journal.pone.0313920 (PMC11651560; doi:10.1371/journal.pone.0313920)
Supplement: Attachment — Submitted filename: Response to Reviewers.docx [file pone.0313920.s008.docx]

**Rebuttal letter Response to reviewer**

We thank the Reviewer and the Editor for their appreciation of our manuscript. Below we respond to all the comments on a point-to-point basis.

This pilot randomised study appears well designed it is perhaps too detailed for the patient numbers concerned (see Table 1). As the authors will know, patient numbers in pilot studies are likely to be insufficient to draw firm conclusions.

In accordance with this remark, we have now simplified Table 1 and retained only essential information.

We agree that our sample size may be not sufficient to draw firm conclusion. This point was already outlined in the limitations paragraph, it is now better specified

“The sample may have been too small to draw firm conclusions about the efficacy or absence of efficacy of HT on ACC activity”. L. 275-6

In this respect, a key question is how large a difference between the two treatment arms is likely? Consequently, the information in Table 2 is important but lacks the magnitude of the differences between groups and the confidence interval on these differences. What is required in Table 2, for the ‘Change in catastrophizing score’ for example is, d = Han – Hor = −2.0 – (−1.4) = −0.6, 95% CI −2.4 to +1.2). [Authors please check the calculation].

Thank you for this important point. We have now added the magnitude of the differences between groups by estimating the differences in mean and their confidence intervals (See new Tables 2 and 3).

I also wondered if a better analysis would compare post-intervention values of the Score (rather than the change). This might allow an easier interpretation of the results. The observed difference on this scale could then be checked for any influence **of the corresponding baseline measures using regression methods.**

Thank you for this pertinent suggestion. We have added a table (Table 2) reporting the comparison of the ACC, rumination and catastrophizing values after horticultural therapy and after handiwork. These results show no differences in ACC perfusion between HT and handiwork and slight differences from baseline as explained in the following sentences

“The mean ACC perfusion score after handiwork did not differ from that after HT (Table 2). The rumination and catastrophizing scores after handiwork and HT were also very close (Table 2).” L. 209-11.

We did not run any models, because we did not find any reason for isolating demographic or clinical factor(s) that would explain the results.

I don’t think using a sensitivity analysis (lines 195-196) is useful here. I suggest the associated details are omitted.

We understand your point of view, nevertheless, we preferred to keep the sensitivity analysis because it was planned in order to explore the potential confounding factors. The results of the sensitivity analysis showed that there was no association between sequence, period or activity and ACC perfusion. We added a sentence in the discussion to illustrate the information brought by the sensitivity analysis.

“The absence of effect of sequence, period or activity on ACC perfusion may suggest stability in ACC blood flow, independent of active requirements [27]”. L. 256-8
